# Serum Cystatin 4 combined with routine clinical laboratory indicators: A support vector machine diagnostic model for early screening of gastrointestinal tumors

**DOI:** 10.1371/journal.pone.0353753

**Published:** 2026-09-21

**Authors:** Huijuan Bi, Lina Yin, Wenhao Fang, Jilu Shen

**Affiliations:** 1 Department of Clinical Laboratory, Anhui Provincial Public Health Clinical Center, Hefei, Anhui, China; 2 Department of Clinical Laboratory, The First Affiliated Hospital of Anhui Medical University North District, Hefei, Anhui, China; Dr Anjali Chatterji Regional Research Institute for Homoeopathy, INDIA

## Abstract

Gastrointestinal tumors lack non-invasive early screening tools, with most patients diagnosed at advanced stages. Serum tumor markers combined with machine learning show promise, but single markers have low specificity. This study aimed to construct a non-invasive diagnostic model using multiple laboratory variables. A retrospective study included 214 gastrointestinal tumor patients (observation group) and 130 non-tumor individuals (control group). Thirty-eight laboratory indicators were detected, and seven core variables (HCT, Age, TP, ALB, PLT, WBC, CST4) were identified via feature selection. Eleven machine learning algorithms were used to build models, with performance evaluated by AUC, sensitivity, specificity, calibration curves, and DCA. Serum CST4 levels were significantly higher in the observation group (*P* < 0.001, AUC = 0.706). The SVM model showed the best performance: AUC = 0.85 (95% CI: 0.78–0.92), sensitivity = 95.4% (95% CI: 0.87–0.98), specificity = 61.5% (95% CI: 0.52–0.71), PPV = 80.5% (95% CI: 0.74–0.86), NPV = 88.9% (95% CI: 0.81–0.94) in the test set. Calibration curves demonstrated high consistency (deviation < 5%), and DCA confirmed superior net benefits. SHapley Additive exPlanations (SHAP) analysis identified HCT, CST4, and Age as top contributors. This internally validated tool demonstrated promising preliminary performance for gastrointestinal tumor screening in high-risk populations (sensitivity 95.4%, specificity 61.5%). Due to its modest specificity, it is suitable as an auxiliary risk stratification tool for high-risk individuals but not for general population screening. External prospective multi-center validation is mandatory before clinical application.

## Introduction

Gastrointestinal malignancies, including gastric, colorectal, and esophageal cancers, are a leading cause of cancer mortality globally [1]. Early diagnosis is crucial, as over 60% of patients are diagnosed at advanced stages, resulting in a 5-year survival rate of less than 30% [1,2]. While endoscopy with histopathology is the gold standard, its invasiveness, cost, and requirement for specialized personnel render it unsuitable for large-scale population screening [3,4]. Consequently, there is an urgent need for a non-invasive, accessible, and cost-effective early detection tool.

Gastroscopy combined with histopathological examination is the gold standard for gastrointestinal tumor diagnosis but carries risks of perforation, bleeding, and infection, and is not suitable for large-scale screening [5]. Serum tumor markers are the mainstay of non-invasive screening due to their convenience, but traditional markers (e.g., CEA, CA724, CA50) have high false-positive rates, low specificity, and high false-negative rates [6], leading to poor clinical performance in early screening—especially in differentiating tumors from benign gastrointestinal diseases. Thus, there is an urgent clinical need to identify novel, high-performance biomarkers and develop integrated multi-indicator models to overcome the shortcomings of single-marker screening and match the actual clinical screening demands.

Cystatin 4 (CST4), a member of the cystatin family, inhibits extracellular matrix hydrolysis by binding to cysteine proteases and is involved in the pathogenesis and progression of gastrointestinal tumors [7]. Recent studies have confirmed that CST4 is a novel tumor marker with significantly elevated expression in gastric and colorectal cancer patients [8–10], and it can serve as a clinical prognostic indicator [11,12]. Routine clinical laboratory indicators reflect the body’s metabolic and immune responses to tumorigenesis and benign lesions, and their combination with novel tumor biomarkers is expected to improve screening accuracy.

Machine learning (ML) algorithms have unique advantages in high-dimensional feature extraction and pattern recognition, making them ideal for integrating heterogeneous clinical and laboratory variables to build superior diagnostic models [13]. Unlike traditional statistical methods, machine learning can capture complex non-linear relationships between multiple indicators and improve the accuracy of early tumor screening and differential diagnosis. However, most existing studies either focus on single biomarkers (e.g., CST4 alone [8]) or use healthy populations as the only control group, which deviates from real-world clinical screening scenarios. Few studies have systematically integrated CST4 with routine laboratory indicators—easily accessible in all clinical settings—via multiple machine learning algorithms to develop a screening model based on a clinically relevant control group (benign gastrointestinal disease and healthy population) for primary healthcare institutions.

Therefore, this study aimed to: (1) compare serum CST4 levels and 37 routine laboratory indicators between gastrointestinal tumor patients and a non-tumor control group; (2) identify a core set of predictive variables through consensus feature selection using multiple algorithms; and (3) construct and rigorously evaluate a suite of ML models, with the goal of identifying an optimal, non-invasive diagnostic model for early screening of gastrointestinal tumors that leverages only routinely accessible clinical laboratory data.

## Materials and methods

### Research subjects

This retrospective study was approved by the Institutional Review Board of Anhui Provincial Public Health Clinical Center (Approval No.: PJ-YX2026–038; approved May 6, 2026). Informed consent was waived by the ethics committee due to the retrospective nature of the study, and all data were de-identified to protect patient privacy. The study was conducted in strict accordance with the Declaration of Helsinki and ethical guidelines issued by the National Health Commission of the People’s Republic of China.

The observation group included 214 patients with gastrointestinal tumors (91 gastric cancer, 80 colorectal cancer, 43 esophageal cancer) admitted between January 2022 and June 2025, confirmed by histopathological biopsy (**Supplementary** S1 Table). The observation group consisted of 144 males (67.3%) and 70 females (32.7%), aged 32–81 years (mean 62.1 ± 10.05 years). The control group (n = 130) included 60 healthy individuals and 70 patients with benign gastrointestinal diseases (chronic gastritis, peptic ulcer, intestinal polyps), with 66 males (50.8%) and 64 females (49.2%), aged 40–88 years (mean 50.9 ± 13.30 years). All control subjects had no history of malignant tumors, severe chronic inflammatory disorders, or organ insufficiency, and no subclinical malignant lesions were found via imaging and laboratory examinations. Exclusion criteria: concurrent malignancies, severe hepatic/renal insufficiency, hematologic disorders, recent infectious diseases, and incomplete laboratory test data.

### Sample size adequacy

Sample size adequacy was evaluated using the events-per-variable (EPV) criterion. With 214 tumor cases and 38 initial predictor variables, the EPV was 5.63, meeting the minimum acceptable EPV of 5 for machine learning modeling. After feature selection (7 core variables retained), the EPV increased to 30.57, well above the recommended threshold of 10 for robust model construction, thereby reducing the risk of overfitting and model instability. It should be noted that EPV criteria were originally developed for traditional logistic regression and may not fully capture the sample-size requirements of modern machine-learning algorithms, which employ regularization, ensemble methods, and internal validation to mitigate overfitting. Therefore, learning curve analysis was additionally performed to confirm sample size adequacy (**Supplementary** S1 Fig).

### Data access time for research purposes

De-identified clinical and laboratory data were accessed between January 2022 and June 2025. Authors did not have access to any identifiable participant information, as all data were de-identified by the hospital’s medical record management department.

### Sample collection and indicator testing

All subjects underwent morning fasting venous blood collection (5 mL). After centrifugation (3000 rpm, 10 min), serum was separated and stored at −80°C until detection. Serum CST4 was measured using an ELISA kit (Shanghai Liangrun Biomedical Technology Co., Ltd., certificate number: 20173403280) meeting Clinical and Laboratory Standards Institute (CLSI) quality control standards for quantitative in vitro diagnostic tests. Blood routine parameters (14 items, including WBC, red blood cell count [RBC], HCT, PLT) were analyzed with an automated hematology analyzer (Mindray BC-6800, electrical impedance and laser scattering principle). Biochemical indicators (16 items, including TP, ALB) were measured using an automated biochemical analyzer (Siemens Centaur XPT). Traditional tumor markers (8 items, including alpha-fetoprotein [AFP], CEA) were detected via chemiluminescent immunoassay (Siemens Atellica IM1600). All tests were performed strictly according to instrument and kit instructions; commercial control materials (Roche Diagnostics) were used for quality control, and all test results were within the acceptable quality control range.

### Data processing and model construction

#### Software and tools.

All data processing, model construction, and visualization were performed using Python 3.10 with scikit-learn (1.5.2), XGBoost (1.7.4), LGBM (4.0.0), and SHAP (1.4.6.1). R 4.5.2 with the rms package (8.1−0) and ggplot2 (4.0.0) was used for calibration curve analysis, decision curve analysis (DCA), and correlation heatmap generation.

#### Data preprocessing.

The overall missing data proportion across all 38 indicators and 344 subjects was 2.6%, with no single indicator exceeding 5% missingness. Missing values were imputed using an iterative imputation approach with RandomForestRegressor (default hyperparameters). The regressor settings were as follows:

- n_estimators = 100: 100 decision trees;
- criterion = “squared_error”: squared error as the split criterion;
- max_depth = None: unlimited tree depth;
- min_samples_split = 2: a minimum of 2 samples required to split an internal node;
- min_samples_leaf = 1: a minimum of 1 sample per leaf node;
- max_features = 1.0: all features considered for each split.

The iterative imputation loop was capped at a maximum of 10 iterations. Critically, to eliminate data leakage, imputation was performed separately within the training and testing datasets. The random forest imputation model was fitted exclusively on the training data, and the identical hyperparameters were applied to predict missing values in the test set. Preliminary validation demonstrated that this random forest imputation strategy outperformed group mean imputation, with differences in sensitivity and specificity of less than 0.5 percentage points. The dataset was randomly split into a training set (70%, *n* = 240) and an independent test set (30%, *n* = 104) using stratified random sampling with a fixed random seed (random_state = 42) to preserve the class distribution. To prevent data leakage, missing data imputation was performed strictly and separately within the training and testing datasets. Feature selection (decision tree, random forest, LASSO, RFE), Pearson correlation filtering was then conducted exclusively on the training set. The selected features and scaling parameters derived from the training set were subsequently applied to the test set. The test set remained completely untouched until final model evaluation. A workflow diagram illustrating this pipeline is provided in Supplementary (**Supplementary** S2 Fig).

#### Feature selection.

Feature selection was performed using four algorithms: decision tree, random forest, Lasso regression, and recursive feature elimination (RFE). Core features were selected based on three principles: (1) features identified by all four algorithms were prioritized; (2) features with high ranking in individual algorithms and high co-occurrence frequency across methods were preferentially selected; (3) features with high model importance and clear clinical significance were additionally incorporated as supplementary core features. After determining the initial 8 core features, Pearson correlation analysis was further applied to eliminate features with high multicollinearity (|r|≥0.9), and the final non-redundant features were retained for subsequent model construction. The entire feature selection process was performed exclusively on the training set to prevent data leakage into the model evaluation.

#### Algorithm selection and model training.

Eleven machine learning algorithms were selected: Naive Bayes, K-Nearest Neighbors (KNN), Logistic Regression, Random Forest, Decision Tree, Artificial Neural Network (ANN), Support Vector Machine (SVM), Gradient Boosting, Light Gradient Boosting Machine (LGBM), AdaBoost, and Extreme Gradient Boosting (XGB). The dataset was randomly split into a training set (70%, n = 240) and an independent test set (30%, n = 104). Hyperparameters for each model were optimized using a nested 5-fold cross-validation strategy with ROC-AUC as the scoring metric. For SVM specifically, we employed GridSearchCV with stratified 5-fold CV. The parameter search space included C∈{0.1,10,100} (controlling regularization strength) and kernel∈{'linear','rbf'}. Regarding the gamma parameter: when kernel = ’rbf’, scikit-learn’s SVC automatically sets gamma = ’scale’ as the default; we intentionally fixed gamma = ’scale’ for the RBF kernel based on preliminary experiments, as this data-driven default generally provides robust performance across our feature sets and avoids overfitting from excessive granularity in the gamma search space. When kernel = ’linear’ was selected, gamma is mathematically not applicable and was ignored by the implementation. The final SVM model was instantiated using the optimal *C* and kernel identified by GridSearchCV, with probability = True to enable probability estimates. The same nested CV framework was applied to the remaining 10 classifiers, with detailed search grids and optimal hyperparameters for each fold provided in **Supplementary** S4 Table. For all classifiers, the decision threshold was optimized by maximizing Youden’s J statistic on the training set (or within each CV fold), and the resulting threshold was then applied to the test set to calculate sensitivity, specificity, PPV, and NPV.

### Machine learning model development and validation

This study was designed and reported in accordance with the TRIPOD-AI (Transparent Reporting of a multivariable prediction model for Individual Prognosis Or Diagnosis—AI extension) guidelines. The dataset was randomly split into a training set (70%, *n* = 240) and an independent test set (30%, *n* = 104) using stratified random sampling with a fixed random seed (random_state = 42) to preserve the class distribution. Missing data imputation was performed separately within the training and testing datasets. Feature selection (decision tree, random forest, LASSO, RFE), Pearson correlation filtering, and Min–Max scaling were conducted exclusively on the training set. The selected features and scaling parameters derived from the training set were applied to the test set.Internal validation was performed using stratified 5-fold cross-validation and bootstrap resampling (1,000 iterations) on the training set to estimate optimism-corrected performance. Calibration was assessed using calibration curves, and overfitting was evaluated via learning curve analysis (**Supplementary** S2 Fig). However, external validation on an independent, prospectively recruited cohort from a different center or geographic region was not performed in this study, which is acknowledged as a critical limitation per TRIPOD-AI Item 19a.

#### Model evaluation metrics.

Primary metrics: Area under the ROC curve (AUC), sensitivity, specificity, with 95% confidence intervals (CIs) for AUC calculated via the bootstrap method (1000 resamples). Calibration curves assessed the consistency between predicted probabilities and actual tumor incidence, with absolute deviation calculated in the threshold range 0.2–0.8. Decision curve analysis (DCA) was performed to calculate clinical net benefits (Net Benefit = (True Positives−False Positives)×Threshold Probability/(1−Threshold Probability)/Total Population), with “All treated” and “all untreated” as references. PPV and NPV were calculated directly from the confusion matrix in the test set (observed prevalence = 62.2%). For projected performance in different prevalence scenarios, we used Bayesian updating:


$$\begin{array}{ccc} \text{PPV} & = & \frac{\text{Sensitivity}\times \text{Prevalence}}{\text{Sensitivity}\times \text{Prevalence}+(1-\text{Specificity})\times (1-\text{Prevalence})}, \end{array}$$ (1)

$$\begin{array}{ccc} \text{NPV} & = & \frac{\text{Specificity}\times (1-\text{Prevalence})}{\left(1-\text{Sensitivity})\times \text{Prevalence}+\text{Specificity}\times (1-\text{Prevalence}\right)}. \end{array}$$ (2)

Projected PPV and NPV were calculated at gastrointestinal tumor prevalence rates of 5%, 10%, and 15% for high-risk populations. SHAP (SHapley Additive exPlanations) analysis was used to assess feature contribution of the optimal model, with summary plots and bar plots generated for visualization.

### Statistical analysis

Data analysis was performed using SPSS 26.0 software and Python 3.10. Continuous variables were expressed as mean±standard deviation (normally distributed) or median (interquartile range, IQR) (non-normally distributed), and compared using independent samples *t*-test or Wilcoxon rank sum test. Categorical variables were expressed as counts (percentages) and compared using the chi-square test. Because 38 biomarkers were compared between groups, both Bonferroni correction and Benjamini-Hochberg false discovery rate (BH-FDR) procedure were applied to control for multiple comparisons. Adjusted *p*-values were reported alongside raw *p*-values, and an adjusted *p*-value <0.05 was considered statistically significant for these comparisons. Statistical significance was defined as *P* < 0.05 for all other analyses. Univariate comparison *p*-values were descriptive and not used for variable selection in the machine learning pipeline.

## Results

### Comparison of test indicators between the two groups

**Fig 1:**
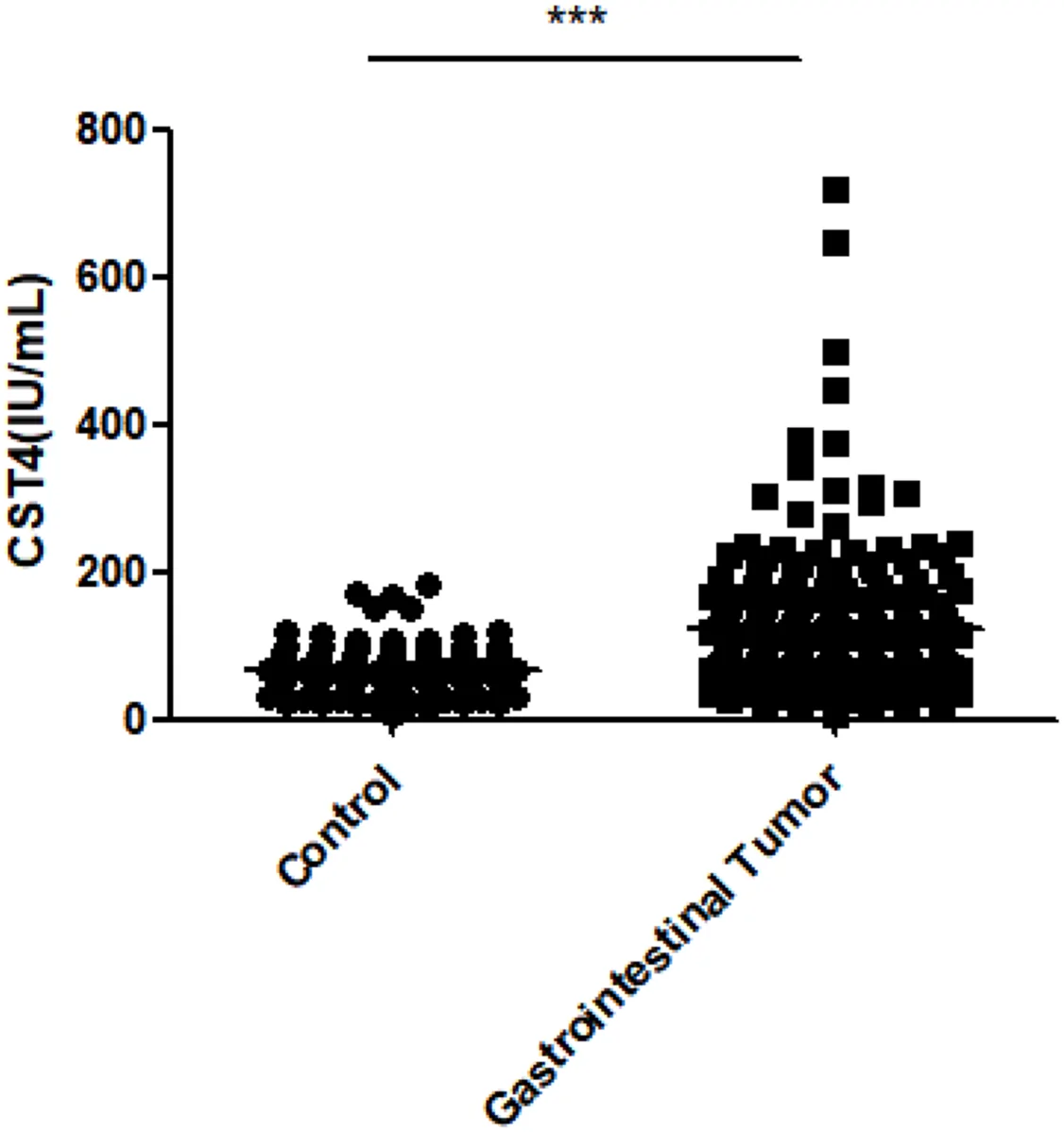
Serum CST4 expression in the gastrointestinal tumor group and the combined control group. The horizontal line represents the median, the box represents the interquartile range, and whiskers represent the range of non-outlier values. ***P < 0.001 vs. control group.

**Fig 2:**
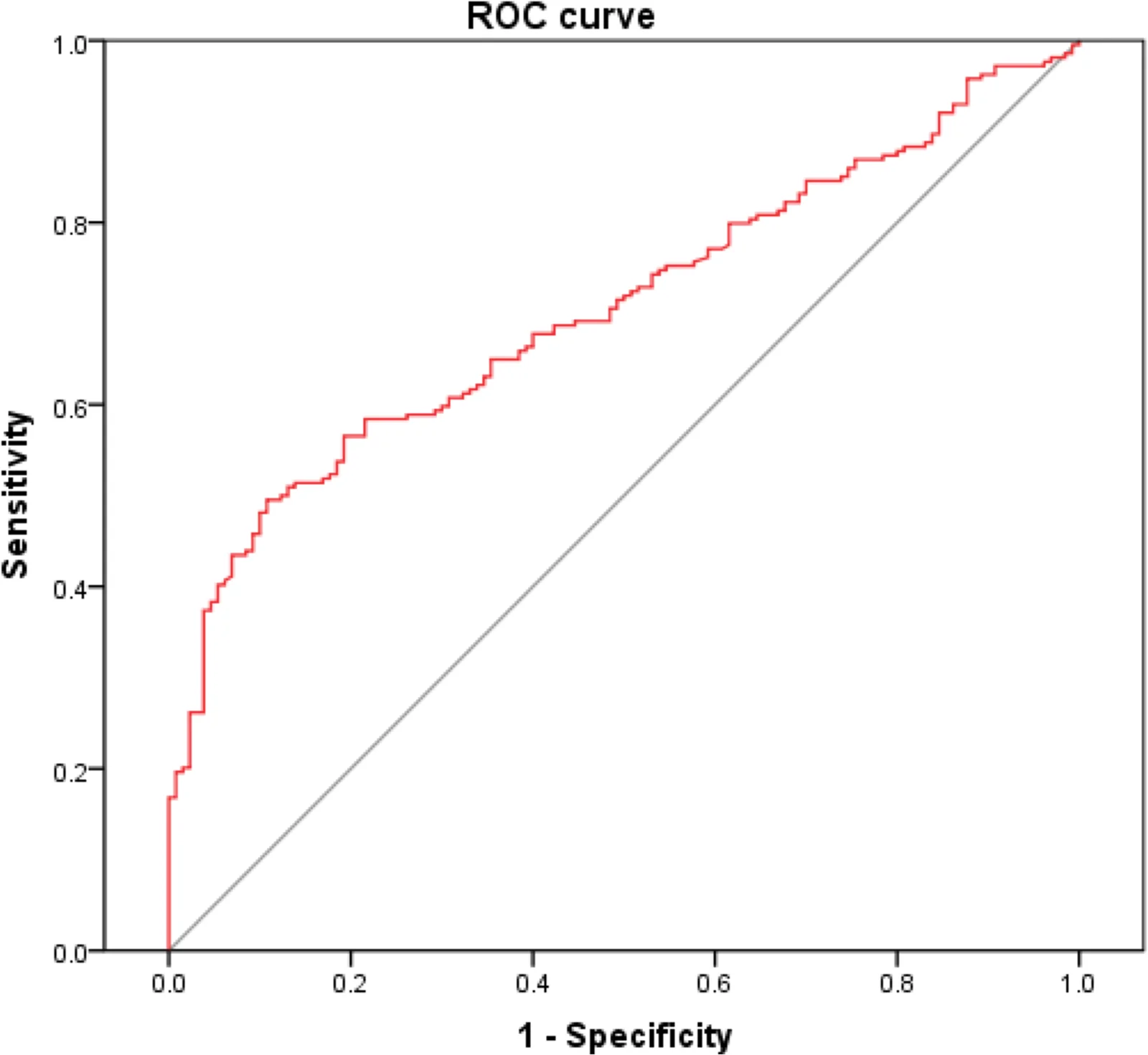
ROC curve of serum CST4 for diagnosing gastrointestinal tumors. AUC = 0.706, 95 percent CI: 0.652-0.759.

Serum CST4 levels in the observation group were significantly higher than in the control group (98.72 [59.83,154.22] vs. 65.12 [48.30,84.25] ng/mL, *P* < 0.001, Fig 1), with an AUC of 0.706 (95% CI: 0.64–0.77) for gastrointestinal tumor diagnosis (Fig 2). Statistical analysis revealed significant differences between the two groups in 10 blood routine parameters, 8 biochemical indicators, and 4 tumor markers (*P* < 0.05, Table 1).

**Table 1:** Comparison of 38 clinical test results between the gastrointestinal tumor group and the control group.

Inspecting item	Gastrointestinal tumor group	Control group	Statistical method	Raw *p*-value	Bonferroni adjusted *p*	BH-FDR adjusted *p*
**Blood routine**						
WBC ($\times 10^{9}$/L)	5.11±1.96	5.95±1.98	Independent samples *t*-test	<0.001	0.019	0.001
NEU ($\times 10^{9}$/L)	2.83 (2.15, 3.82)	3.13 (2.68, 4.10)	Wilcoxon rank sum test	<0.05	0.950	0.041
LYM ($\times 10^{9}$/L)	1.43±0.60	1.74±0.56	Independent samples *t*-test	<0.001	0.019	0.001
MON ($\times 10^{9}$/L)	0.35 (0.27, 0.44)	0.33 (0.28, 0.41)	Wilcoxon rank sum test	0.28	1.000	0.343
EOS ($\times 10^{9}$/L)	0.07 (0.03, 0.14)	0.10 (0.06, 0.17)	Wilcoxon rank sum test	<0.05	0.950	0.041
BAS ($\times 10^{9}$/L)	0.02 (0.01, 0.03)	0.02 (0.01, 0.03)	Wilcoxon rank sum test	0.82	1.000	1.000
RBC ($\times 10^{12}$/L)	3.58±0.66	4.43±0.55	Independent samples *t*-test	<0.001	0.019	0.001
HGB (g/L)	107.12±18.25	132.82±17.61	Independent samples *t*-test	<0.001	0.019	0.001
HCT (%)	33.07±5.50	40.55±4.98	Independent samples *t*-test	<0.001	0.019	0.001
MCV (fL)	94.10 (88.78, 97.70)	92.30 (89.28, 94.45)	Wilcoxon rank sum test	<0.05	0.950	0.041
MCH (pg)	30.40 (28.70, 31.90)	30.20 (29.30, 31.08)	Wilcoxon rank sum test	0.34	1.000	0.392
MCHC (g/L)	323.00 (318.00, 328.75)	328.00 (322.00, 332.75)	Wilcoxon rank sum test	<0.001	0.019	0.001
PCT ($\times 10^{9}$/L)	188.28±78.78	211.10±55.76	Independent samples *t*-test	<0.05	0.950	0.041
RET (%)	0.04 (0.03, 0.06)	0.04 (0.04, 0.05)	Wilcoxon rank sum test	0.30	1.000	0.356
**Biochemical indicators**						
TP (g/L)	61.05 (57.42, 64.18)	65.80 (63.37, 69.40)	Wilcoxon rank sum test	<0.001	0.019	0.001
ALB (g/L)	36.30 (33.40, 39.20)	41.45 (38.42, 44.10)	Wilcoxon rank sum test	<0.001	0.019	0.001
GLOB (g/L)	24.60 (21.95, 27.37)	24.60 (22.22, 27.65)	Wilcoxon rank sum test	0.67	1.000	0.707
TBA (μmol/L)	4.66 (2.00, 8.50)	3.70 (2.70, 5.47)	Wilcoxon rank sum test	0.072	1.000	0.114
TBIL (μmol/L)	10.60 (8.64, 14.36)	13.52 (10.83, 17.49)	Wilcoxon rank sum test	<0.001	0.019	0.001
DBIL (μmol/L)	3.61 (2.82, 5.20)	4.14 (3.25, 5.46)	Wilcoxon rank sum test	<0.05	0.950	0.041
IBIL (μmol/L)	7.37 (5.64, 9.45)	9.48 (7.60, 11.98)	Wilcoxon rank sum test	<0.001	0.019	0.001
ALT (U/L)	17.00 (12.00, 23.00)	17.00 (13.00, 25.00)	Wilcoxon rank sum test	0.27	1.000	0.342
AST (U/L)	19.00 (16.00, 24.00)	17.94 (15.00, 22.00)	Wilcoxon rank sum test	0.081	1.000	0.123
LDH (U/L)	163.50 (142.25, 195.00)	167.65 (155.40, 182.57)	Wilcoxon rank sum test	0.46	1.000	0.499
ALP (U/L)	72.00 (60.00, 91.00)	69.00 (62.02, 80.92)	Wilcoxon rank sum test	0.17	1.000	0.231
BUN (mmol/L)	5.65 (4.71, 6.76)	5.03 (4.19, 6.12)	Wilcoxon rank sum test	<0.05	0.950	0.041
CREA (μmol/L)	60.00 (46.90, 69.78)	55.25 (46.08, 65.35)	Wilcoxon rank sum test	0.15	1.000	0.211
UA (μmol/L)	285.00 (240.25, 340.00)	287.00 (240.10, 353.50)	Wilcoxon rank sum test	0.82	1.000	0.842
CHOL (mmol/L)	3.85 (3.30, 4.46)	4.37 (3.82, 4.92)	Wilcoxon rank sum test	<0.001	0.019	0.001
TG (mmol/L)	0.97 (0.79, 1.33)	1.14 (0.85, 1.52)	Wilcoxon rank sum test	<0.05	0.950	0.041
**Tumor markers**						
AFP (ng/mL)	2.73 (2.02, 3.55)	2.52 (1.70, 3.27)	Wilcoxon rank sum test	<0.05	0.950	0.041
CEA (ng/mL)	1.46 (0.71, 2.48)	0.80 (0.50, 1.60)	Wilcoxon rank sum test	<0.001	0.019	0.001
CA19−9 (U/mL)	7.14 (1.69, 14.75)	5.06 (2.38, 11.67)	Wilcoxon rank sum test	0.11	1.000	0.161
CA125 (U/mL)	7.64 (5.15, 12.50)	7.79 (5.16, 10.27)	Wilcoxon rank sum test	0.27	1.000	0.342
CA72−4 (U/mL)	2.69 (1.18, 7.02)	1.96 (1.04, 4.18)	Wilcoxon rank sum test	<0.05	0.950	0.041
CA50 (U/mL)	6.84 (4.51, 10.93)	5.70 (3.21, 8.09)	Wilcoxon rank sum test	<0.001	0.019	0.001
CA242 (U/mL)	3.81 (2.11, 6.91)	3.66 (1.83, 6.29)	Wilcoxon rank sum test	0.40	1.000	0.447
CST4 (ng/mL)	98.72 (59.83, 154.22)	65.12 (48.30, 84.25)	Wilcoxon rank sum test	<0.001	0.019	0.001

*Notes.* Continuous data are presented as mean ± standard deviation for normally distributed variables, and as median (interquartile range) for non-normally distributed variables. Between-group comparisons were performed using independent samples *t*-test for normally distributed data, and Wilcoxon rank-sum test for non-normally distributed data. All *p*-values are two-sided. To account for multiple comparisons across 38 biomarkers, we applied both the Bonferroni correction and the Benjamini-Hochberg false discovery rate (BH-FDR) procedure. An adjusted *p*-value < 0.05 was considered statistically significant.

*Abbreviations.* WBC, white blood cell count; NEU, neutrophil count; LYM, lymphocyte count; MON, monocyte count; EOS, eosinophil count; BAS, basophil count; RBC, red blood cell count; HGB, hemoglobin; HCT, hematocrit; MCV, mean corpuscular volume; MCH, mean corpuscular hemoglobin; MCHC, mean corpuscular hemoglobin concentration; PCT, plateletcrit; RET, reticulocyte percentage; TP, total protein; ALB, albumin; GLOB, globulin; TBA, total bile acid; TBIL, total bilirubin; DBIL, direct bilirubin; IBIL, indirect bilirubin; ALT, alanine aminotransferase; AST, aspartate aminotransferase; LDH, lactate dehydrogenase; ALP, alkaline phosphatase; BUN, blood urea nitrogen; CREA, creatinine; UA, uric acid; CHOL, total cholesterol; TG, triglyceride; AFP, alpha-fetoprotein; CEA, carcinoembryonic antigen; CA19−9, carbohydrate antigen 19−9; CA125, carbohydrate antigen 125; CA72−4, carbohydrate antigen 72−4; CA50, carbohydrate antigen 50; CA242, carbohydrate antigen 242; CST4, cystatin S4.

### Results of feature variable screening

**Fig 3:**
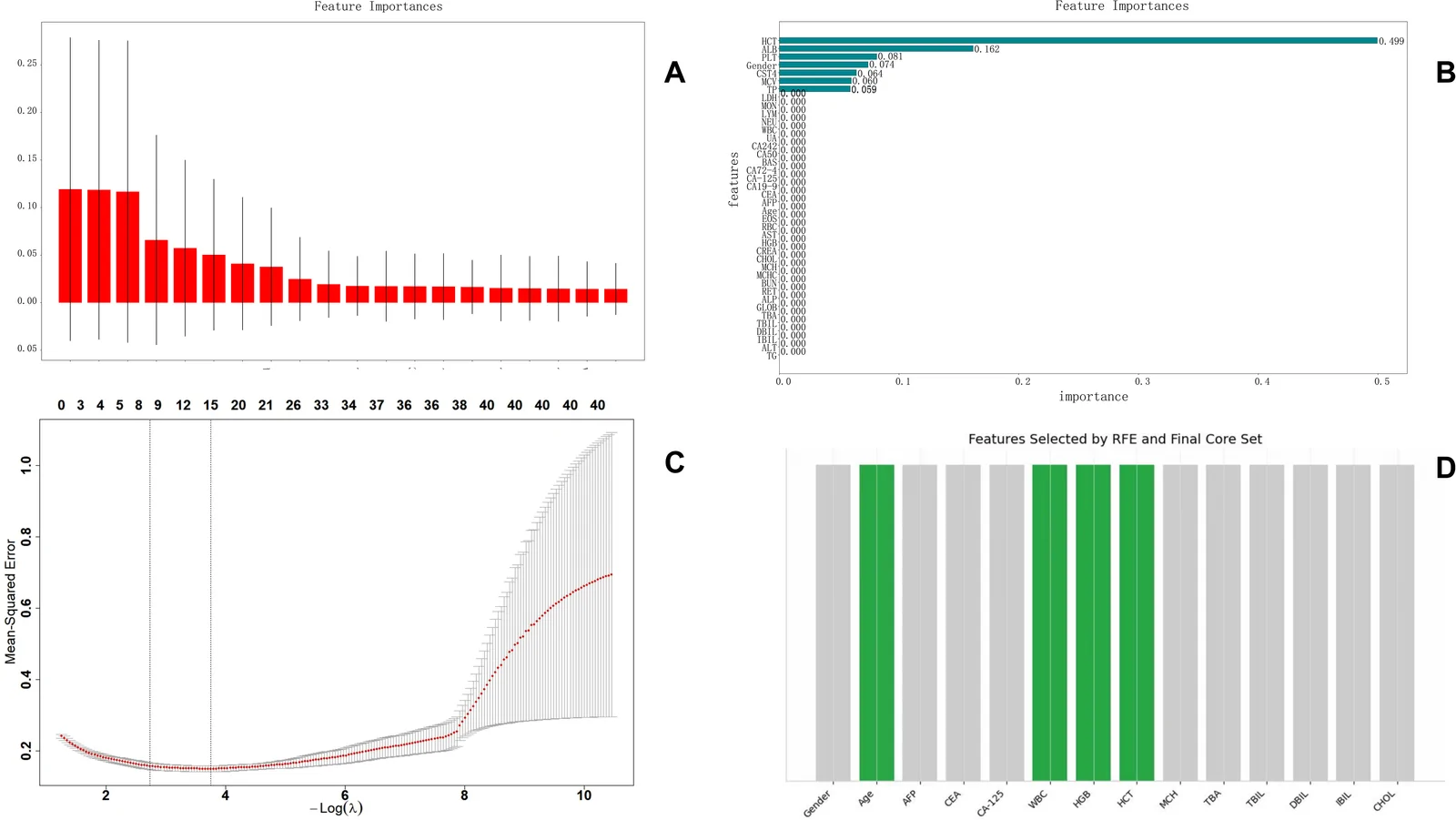
Feature selection results from three machine learning algorithms. (A) Feature importance plot from the random forest model, with error bars indicating the variability of importance estimates. HCT, red blood cell count (RBC), and hemoglobin (HGB) are ranked as the top three most important features. (B) Feature importance plot from the decision tree model. Features are ranked by decreasing importance, and the top 7 features with non-zero contributions are displayed. Hematocrit (HCT) shows the highest importance (0.499), followed by albumin (ALB, 0.162) and platelet count (PLT, 0.081).(C) Lasso regression coefficient path plot. The x-axis represents −log(λ), and the y-axis represents the mean squared error (MSE). The vertical dashed lines indicate the optimal λ values, corresponding to the number of features retained (9–12) that balance model complexity and predictive performance. (D) Feature selection results by Recursive Feature Elimination (RFE). A total of 14 features were retained. Green bars indicate features that were also included in the final 8 core features.

**Fig 4:**
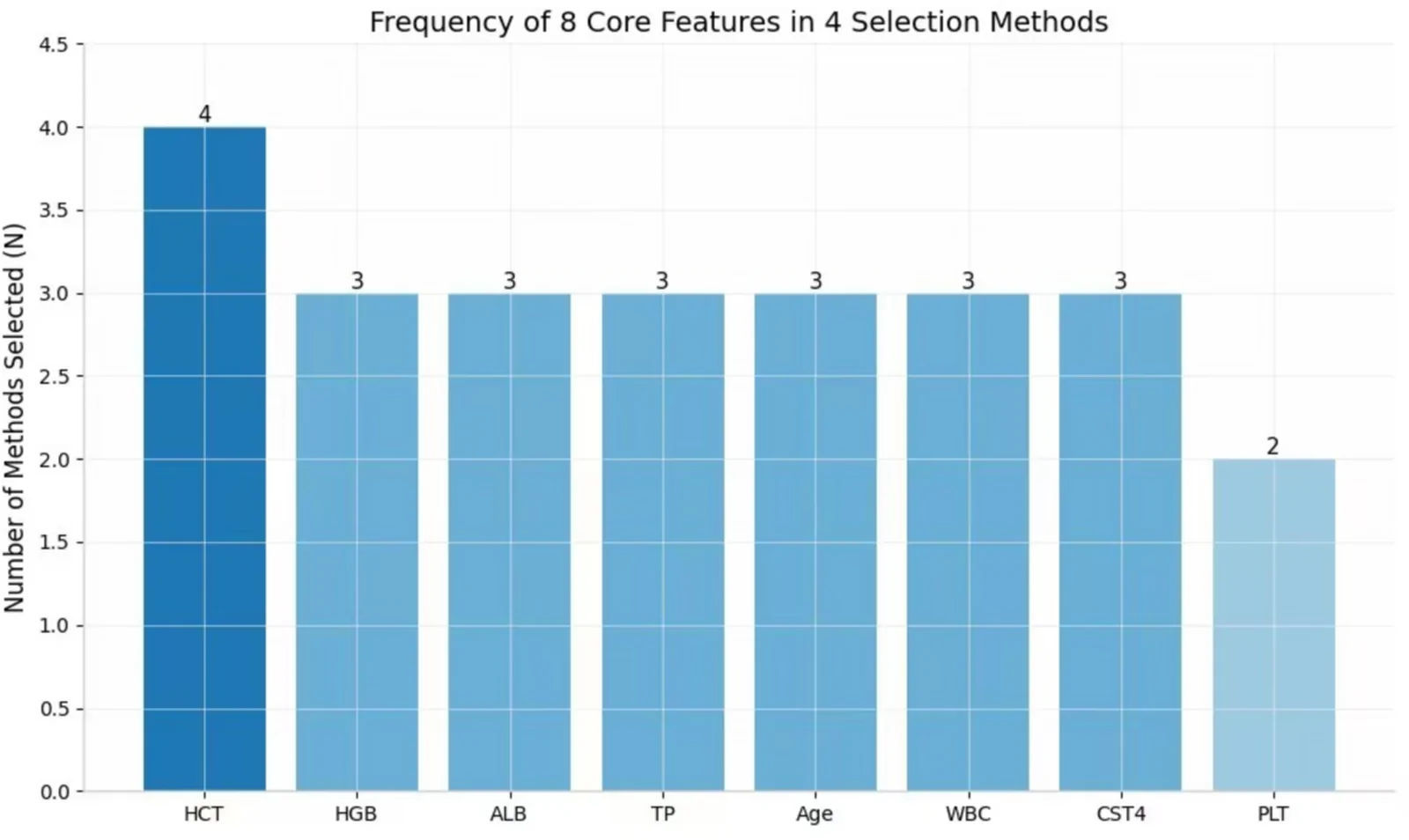
Selection of the 8 core feature parameters based on four feature selection methods. The bar chart shows the co-occurrence frequency of the 8 final selected features across four methods (random forest, decision tree, recursive feature elimination [RFE], and Lasso regression). The y-axis represents the number of methods in which each feature was selected. Features with higher co-occurrence frequencies were prioritized; HCT was selected by all 4 methods, while HGB, ALB, TP, Age, WBC, and CST4 were selected by 3 methods. Notably, PLT was included in the final 8 candidate features due to its high importance ranking in the decision tree model, despite being selected by only 2 of the 4 methods.

**Fig 5:**
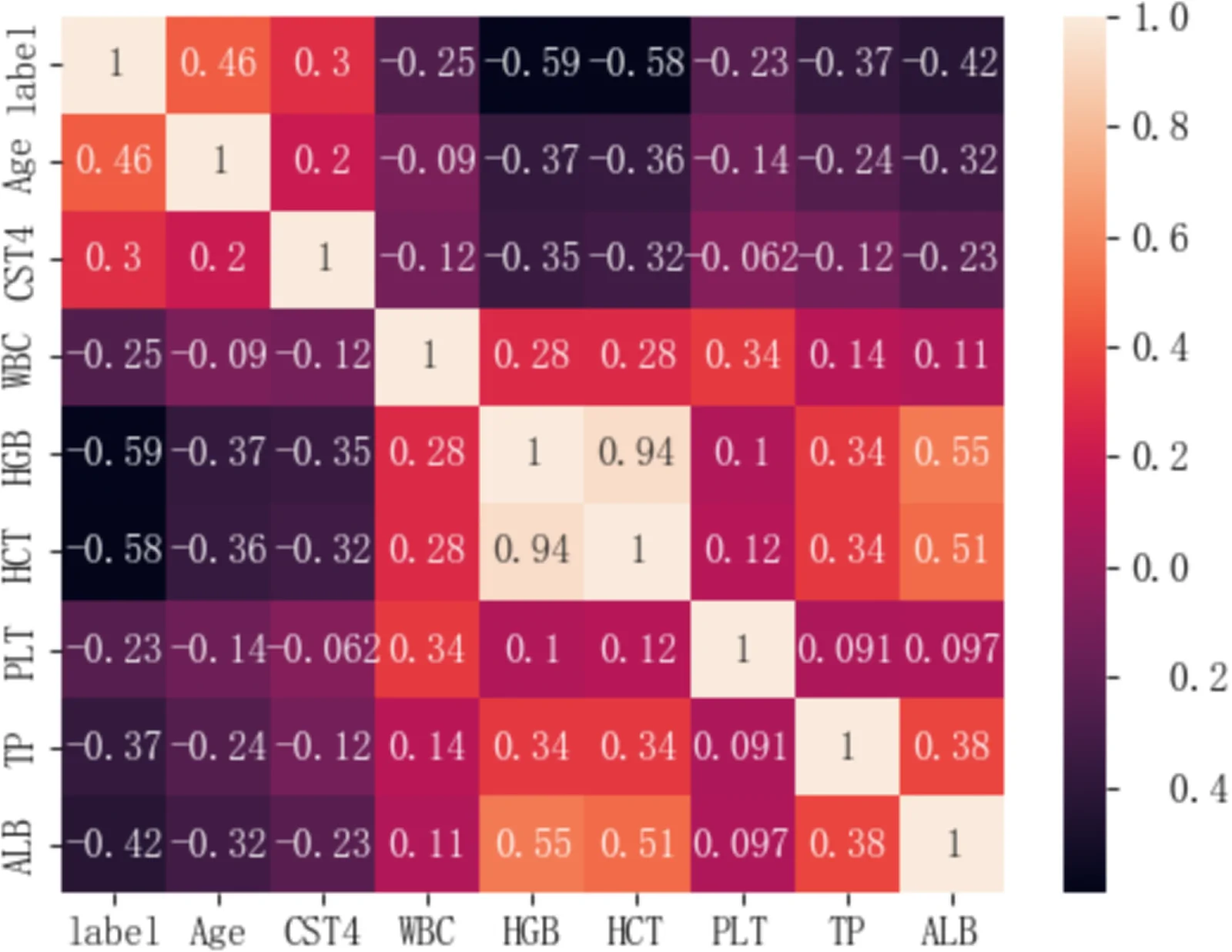
Pearson correlation heatmap of potential feature variables. The color depth represents the correlation coefficient; red indicates positive correlation, blue indicates negative correlation. HGB and HCT show a strong positive correlation (*r* = 0.94).

Four feature selection algorithms (random forest, Fig 3A; decision tree, Fig 3B; LASSO regression, Fig 3C; RFE, Fig 3D) were used to evaluate variable importance. Based on selection frequency and ranking, 8 candidate features (HCT, HGB, Age, TP, ALB, PLT, CST4, WBC) were determined (Fig 4). Pearson correlation analysis showed a strong correlation between HGB and HCT (*r* = 0.94, Fig 5), so HGB was excluded to avoid collinearity. Finally, seven core variables were confirmed: HCT, Age, TP, ALB, PLT, CST4, WBC.

### Comparison of diagnostic performance of different machine learning algorithms

**Fig 6:**
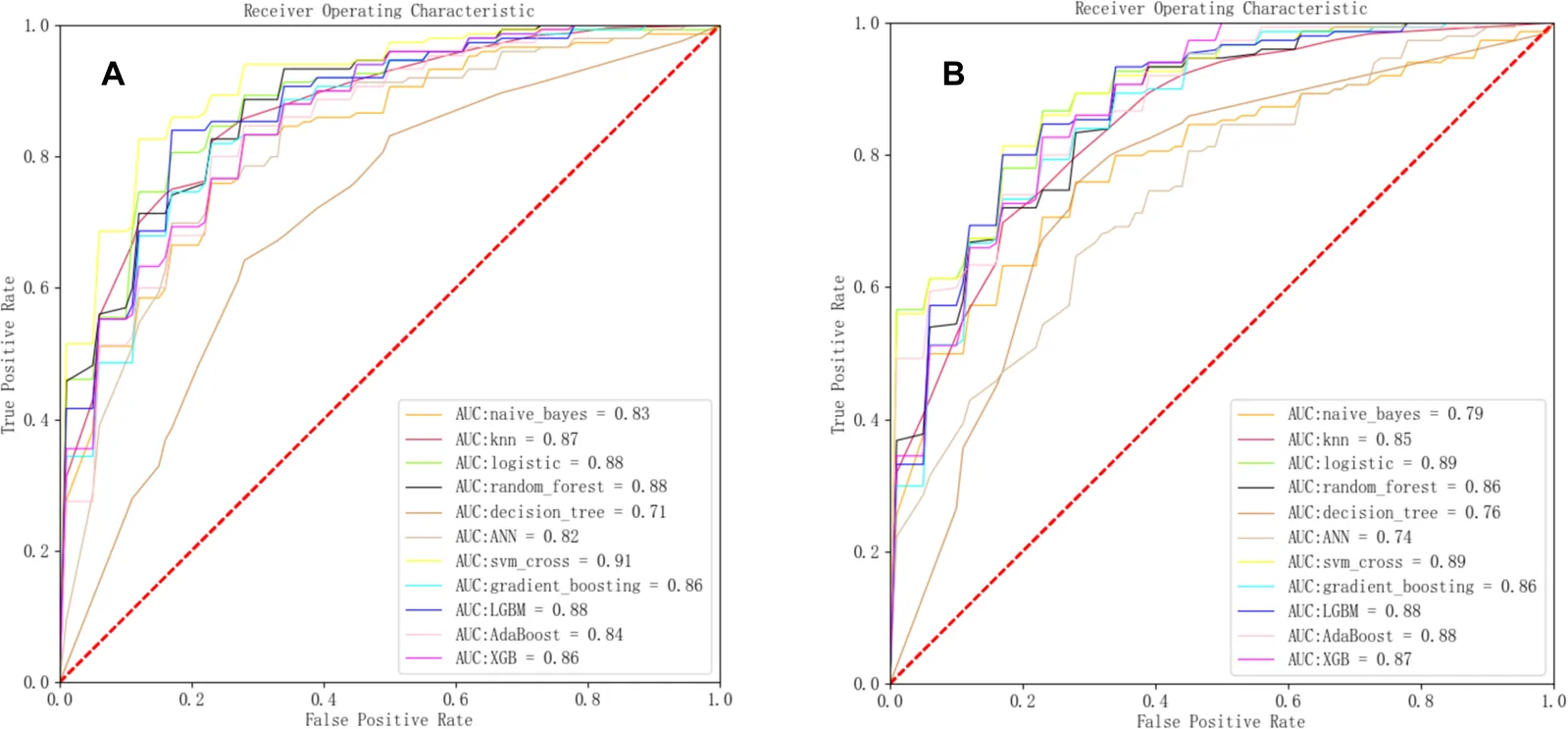
ROC curves of 11 machine learning models. A: Before feature screening; B: After feature screening. The red dashed line represents the random classification reference line (AUC = 0.5). The AUC values for each model are provided in the legend.

**Fig 7:**
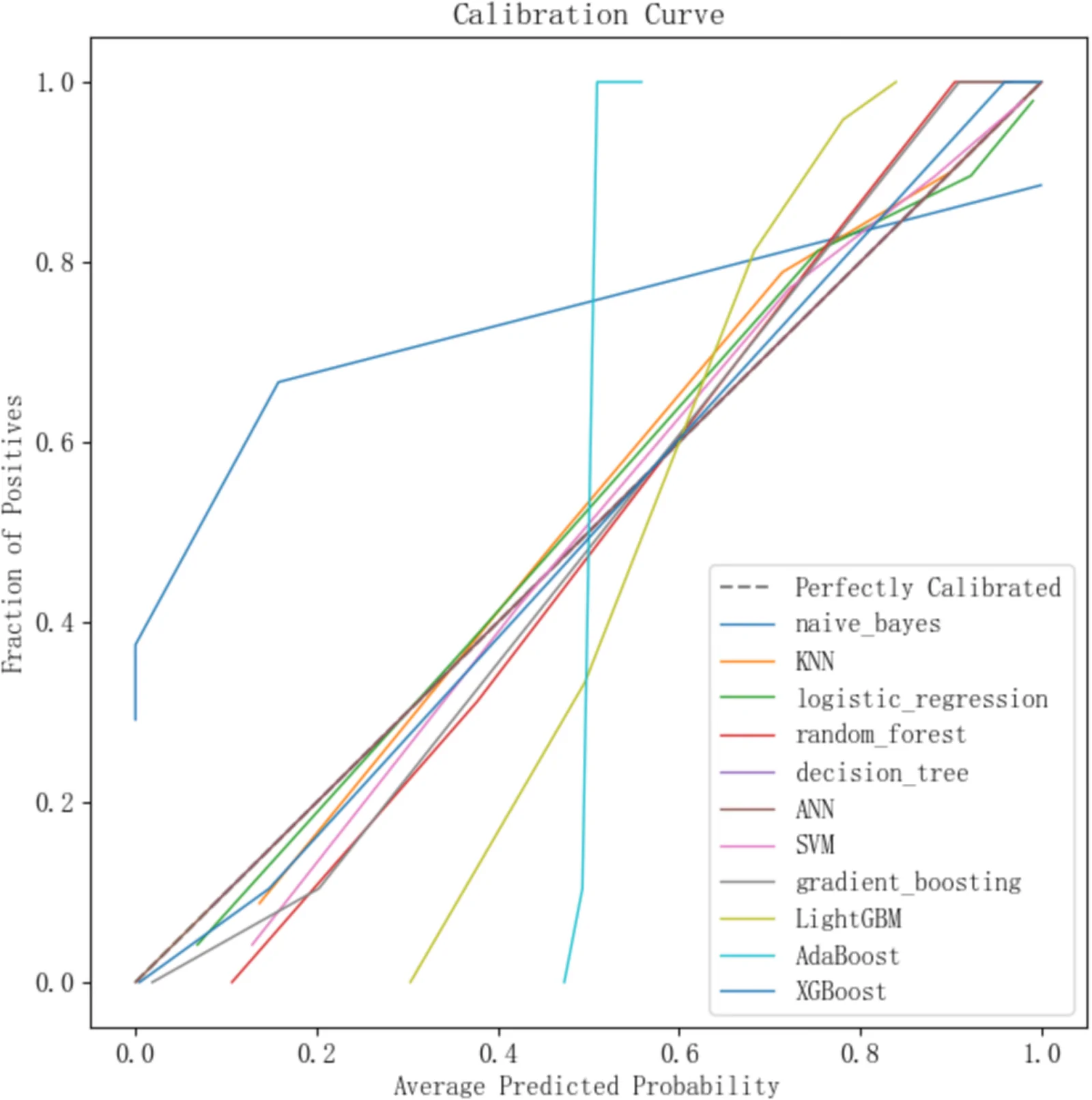
Calibration curves of 11 machine learning models. The dashed line represents the ideal calibration line; the pink curve (SVM) shows the best fit, indicating high consistency between predicted probabilities and actual incidence.

In the training set (5-fold cross-validation), all models except Naive Bayes achieved AUC > 0.8 (Fig 6A), and model performance remained robust after feature reduction (Fig 6B). Calibration curves showed that SVM, Random Forest, and Logistic Regression had the best fit to the ideal calibration line, with SVM performing the best (Fig 7). While this suggests limited evidence of overfitting, calibration performance alone is insufficient to definitively exclude overfitting. Learning curve analysis further supported this by showing converging training and validation AUCs with increasing sample size, indicating adequate training data (*n* = 240) and minimal overfitting for the 7-feature SVM model (**Supplementary** S1 Fig). Based on comprehensive performance (AUC, calibration, clinical net benefit), the SVM model was selected as the optimal algorithm.

### Verification of model generalization ability in the test set

**Fig 8:**
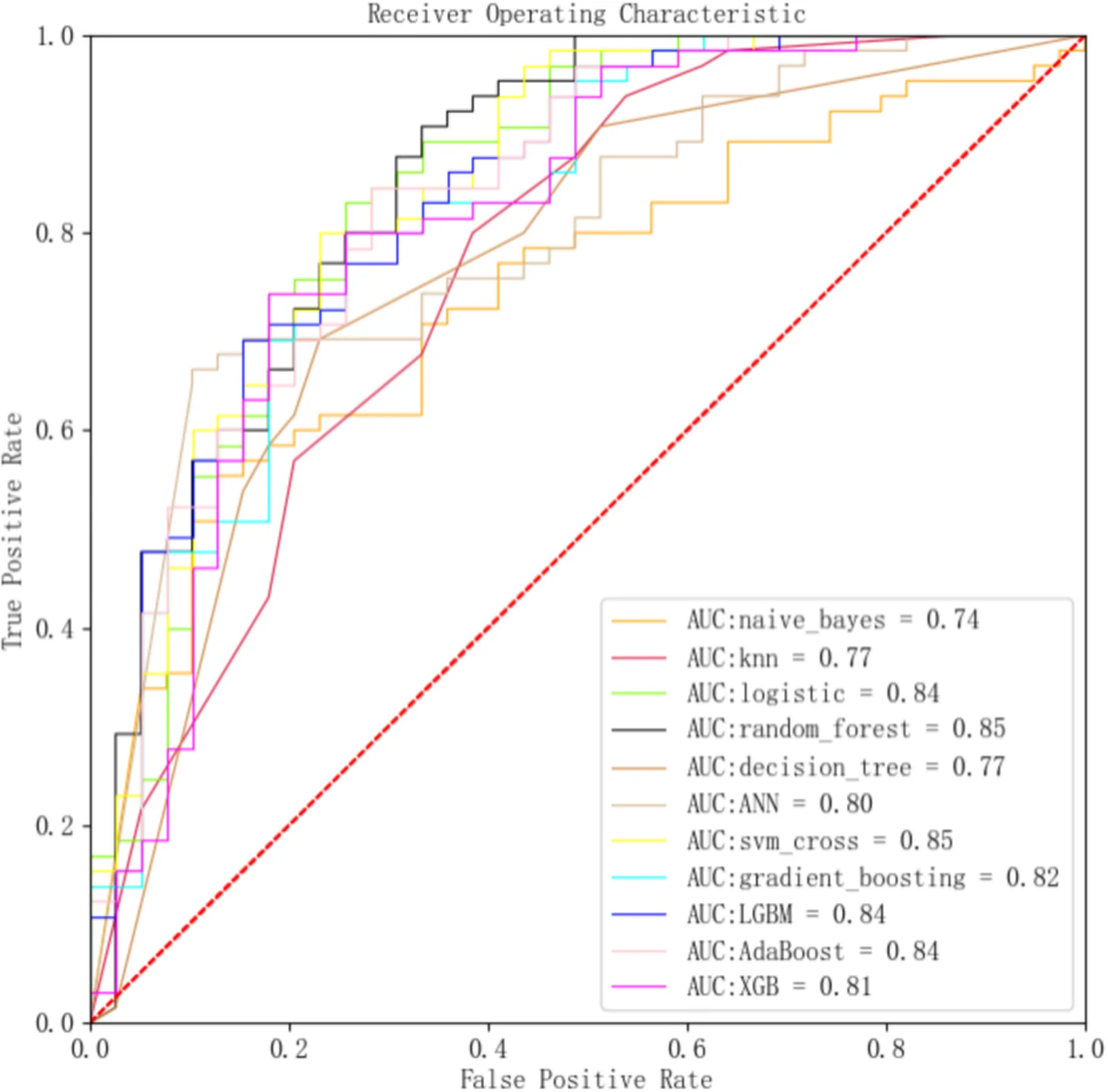
ROC curves of 11 machine learning models in the test set. The yellow curve (SVM) has an AUC of 0.85, showing excellent generalization ability.

**Fig 9:**
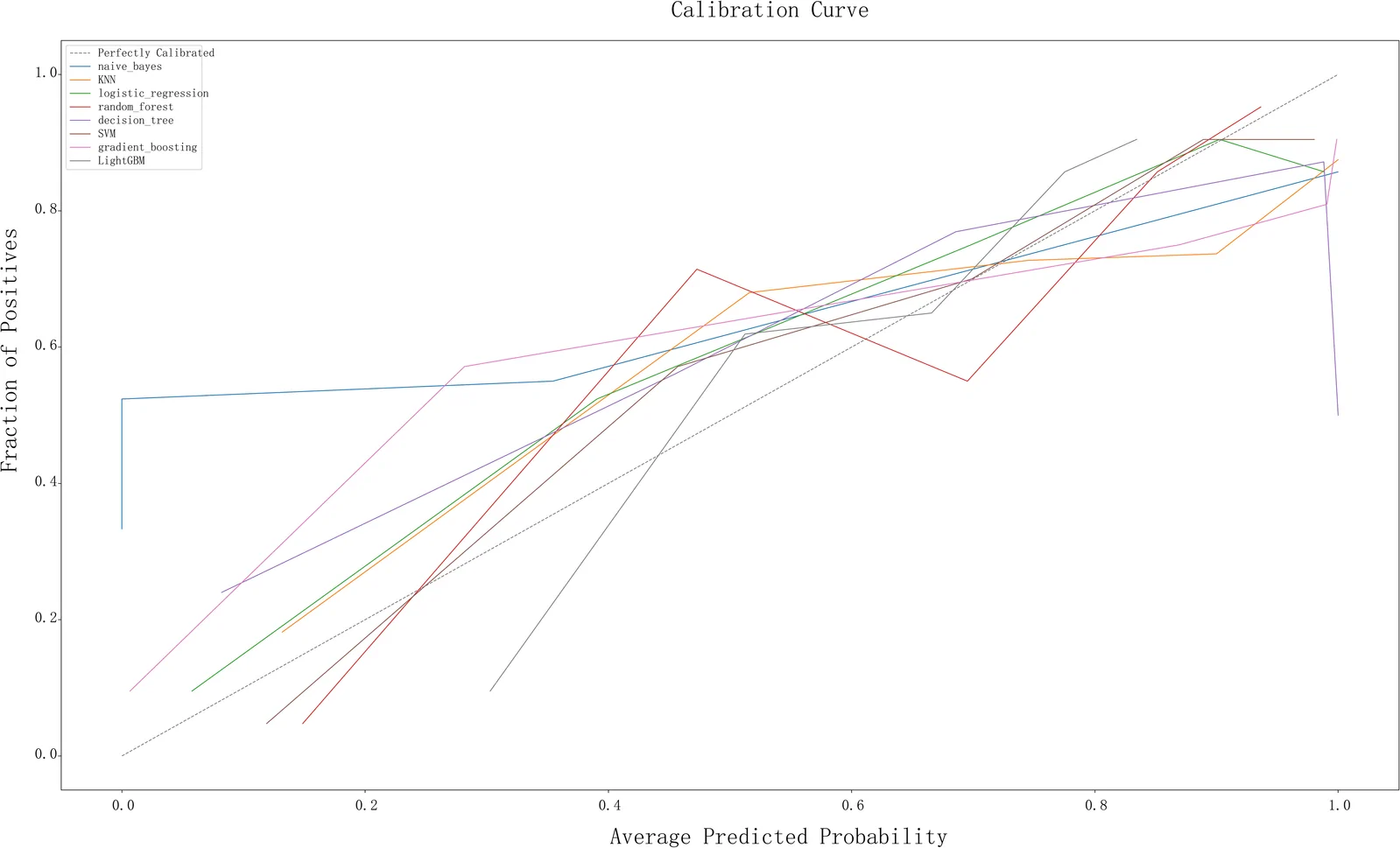
Calibration curve of the test set. The pink curve (SVM) is almost overlapping with the ideal calibration line (dashed line) in the threshold range of 0.2–0.8.

**Fig 10:**
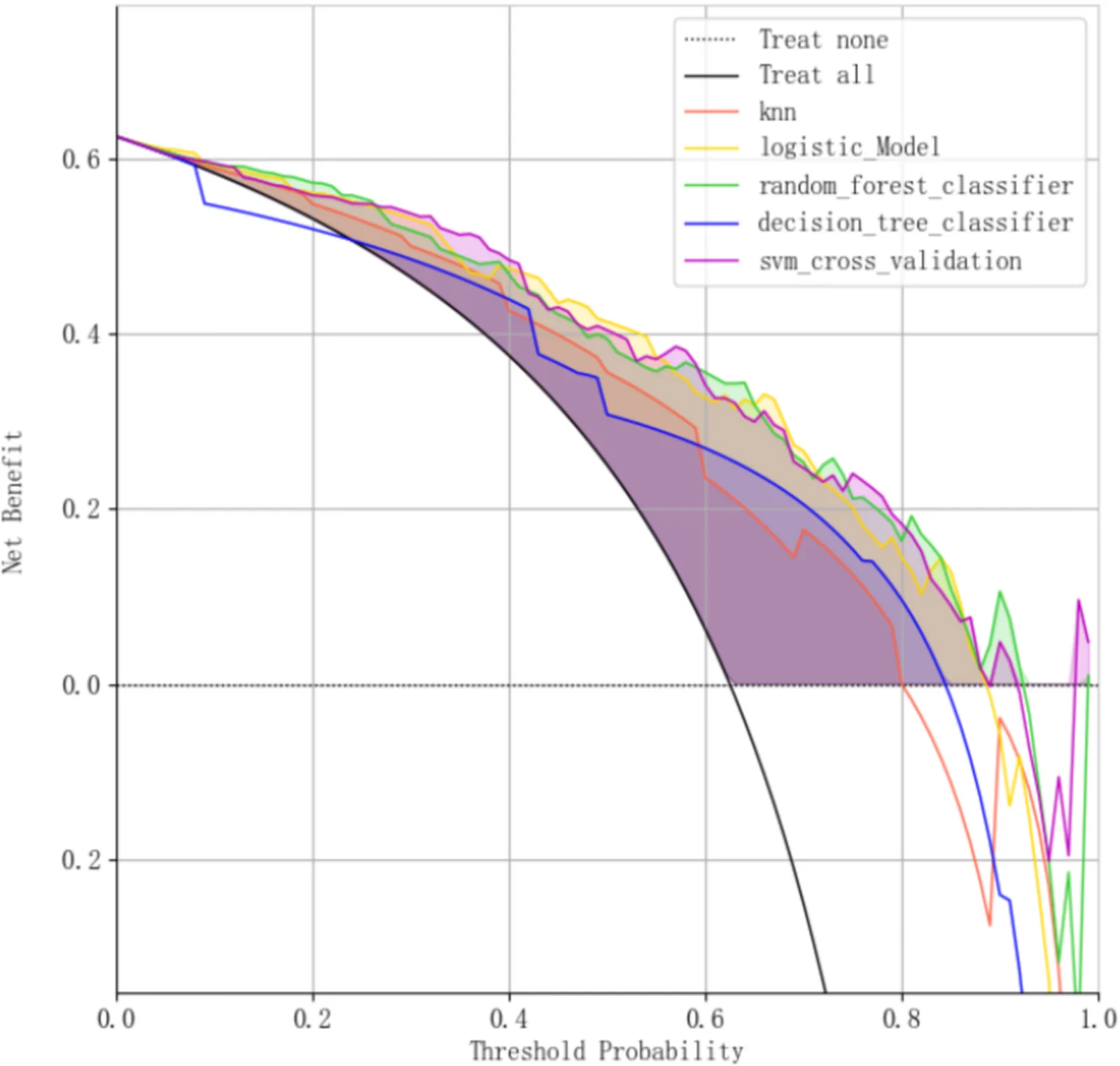
DCA curve of the test set. The red curve (SVM) shows higher net benefits than other algorithms and reference strategies (gray dashed line: “all untreated”; black curve: “all treated”) within the threshold probability of 0.1–0.8.

The SVM model yielded an AUC of 0.85 in the test set (Fig 8), with a sensitivity of 95.4%, a specificity of 61.5%, a PPV of 80.5 percent, and a NPV of 88.9% at the observed disease prevalence of 62.2%. Projected performance of the model in different prevalence scenarios is summarized in S2 Table. To ensure that the model performance was not driven by age or sex imbalances, we performed age- and sex-matched analysis and age-stratified analysis (S3 Table). After matching, the model achieved an AUC of 0.84 (95% CI 0.76–0.91), with a negligible decrease of −0.010 compared with the original model, confirming that the discriminative ability was independent of demographic imbalances. Stratified analysis revealed acceptable performance in the < 55 years group [AUC 0.89 (95% CI 0.76–1.00)] and the 55–65 years group [AUC 0.86 (95% CI 0.72–0.99)], but poor discrimination in the > 65 years group [AUC 0.58 (95% CI 0.36–0.80)], suggesting limited applicability in elderly populations. Sensitivity analysis excluding age yielded an AUC of 0.86 (95% CI 0.78–0.93), indicating a minimal contribution of age to model discrimination. Calibration curve analysis showed the SVM model’s predicted probabilities were highly consistent with actual tumor incidence, with a deviation < 5% in the threshold range 0.2–0.8 (Fig 9). DCA indicated the SVM model’s net benefit was significantly higher than other algorithms and the “all treated” or “all untreated” strategies within the threshold probability range of 0.1–0.8 (Fig 10).

### SHAP feature contribution analysis

**Fig 11:**
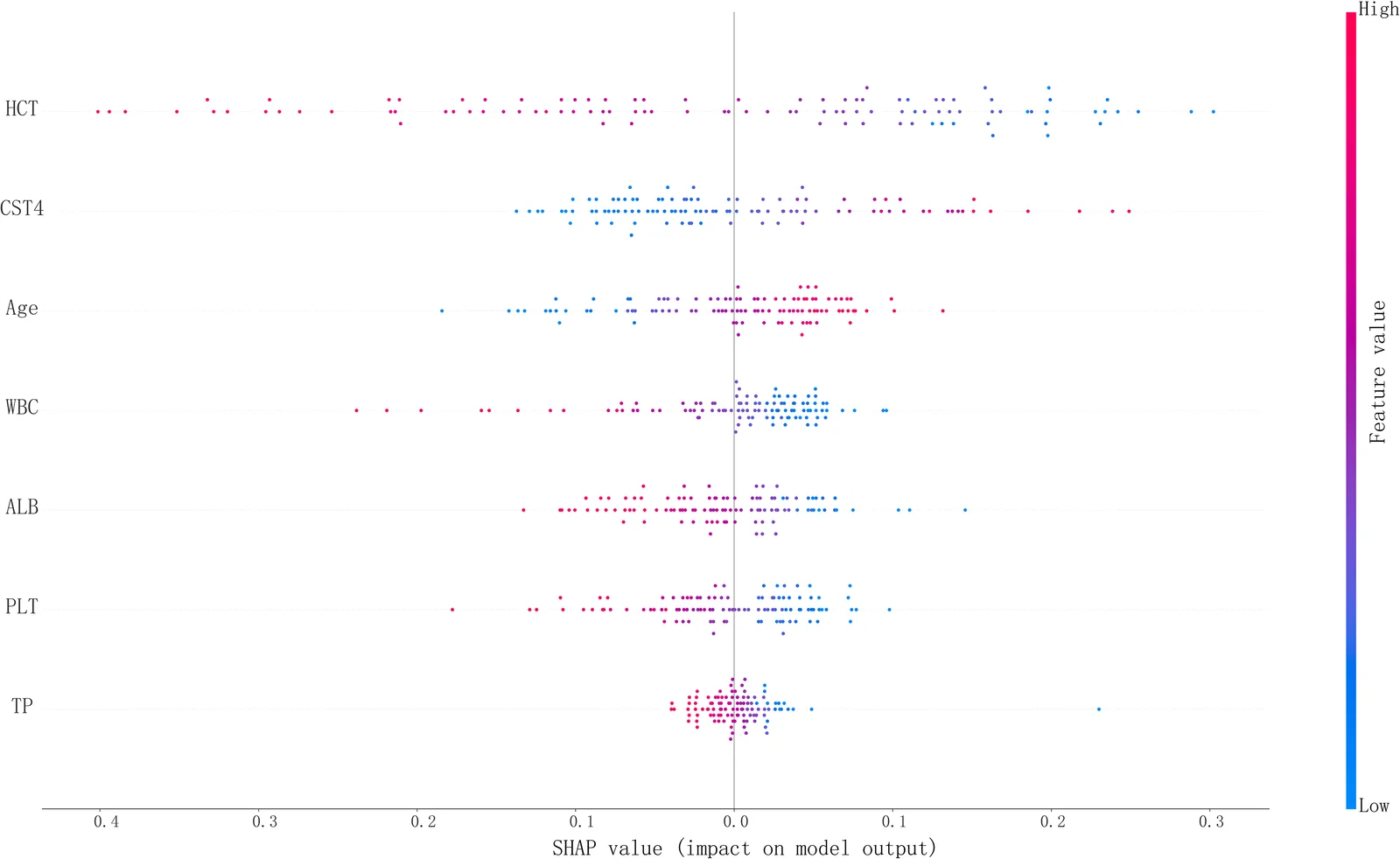
SHAP summary plot of the SVM model. Each row represents a core feature; the abscissa is the SHAP value (positive values promote tumor prediction, negative values inhibit it). Red dots indicate higher feature values, blue dots indicate lower feature values.

SHAP analysis revealed that HCT, CST4, and age were the top positive contributors to the SVM model (Fig 11), with HCT having the widest SHAP value distribution, indicating the most significant impact on diagnostic outcomes. WBC, ALB, and PLT were negative contributors, while TP had a negligible impact (SHAP value ≈0).

## Discussion

Early diagnosis is crucial for improving the prognosis of gastrointestinal tumor patients, as over 60% of cases are diagnosed at intermediate or advanced stages with a 5-year survival rate of less than 30% [3]. Current non-invasive screening relies on traditional serum tumor markers with poor performance in differentiating tumors from benign gastrointestinal diseases, and invasive gold standard methods are not suitable for large-scale screening. This study constructed a non-invasive diagnostic model using seven readily available laboratory indicators and machine learning algorithms and systematically evaluated the model’s performance, interpretability, and clinical applicability, addressing the limitations of single-biomarker screening and traditional case-control studies with only healthy controls, and providing a practical auxiliary tool for primary hospitals.

Our results confirm that serum CST4 is a promising novel biomarker for gastrointestinal tumor screening, with significantly elevated levels in gastrointestinal tumor patients (*P* < 0.001) and an AUC of 0.706 for single-indicator diagnosis. This performance is superior to traditional tumor markers such as CEA (AUC 0.62–0.68 [6]) and CA50 (AUC 0.65–0.70 [6]), consistent with previous studies reporting CST4’s high diagnostic value for digestive system tumors [7,9].

Routine laboratory indicators reflect the body’s systemic response to tumors: HCT, ALB, and TP were significantly lower in the tumor group (*P* < 0.001), likely due to tumor-induced malnutrition and impaired nutrient absorption [14]; WBC and PLT abnormalities may be associated with tumor-related inflammatory responses [15]. Although the combination of these indicators with CST4 led to a mild reduction in specificity, it dramatically enhanced sensitivity to 95.4% from 76.2% with CST4 alone, making it more favorable for cases requiring high detection rates [6]. Notably, all seven core indicators are routinely detected in clinical laboratories, requiring no additional tests or expensive equipment, a critical advantage over previous models relying on specialized biomarkers [7,9].

Machine learning algorithms excel at multi-index integrated analysis [16,17]. This study screened 7 core variables from 38 clinical indicators through Random Forest, Decision Tree, Lasso, and RFE recursive elimination analysis, and constructed models using 11 algorithms, with the SVM model identified as optimal (test set AUC = 0.85, sensitivity = 95.4%, specificity = 61.5%). The observed PPV and NPV reflect the high study prevalence (62.2%); in low-prevalence settings (e.g., 5% in high-risk clinics), PPV decreases to ~11.5%, while NPV remains high, highlighting the model’s value as a rule-out test. However, the specificity of 61.5% implies that ~38.5% of non-tumor individuals would yield false-positive results, necessitating confirmatory endoscopy for a substantial proportion. Future studies should optimize the decision threshold using decision curve analysis or cost-benefit frameworks to better balance sensitivity and specificity for potential clinical implementation after external validation. The model’s 2-hour turnaround time reduces early screening report time by 80% compared with endoscopy, suggesting it may serve as a potentially useful auxiliary tool for high-risk population screening. SVM is suitable for high-dimensional small-sample data, matching this study’s characteristics (38 indicators, 344 samples), and hyperparameter tuning and 5-fold cross-validation improved model robustness. Compared with previous studies focusing on CST4 alone [8] or using only healthy controls [7], this multi-indicator SVM model achieved higher diagnostic accuracy in a clinically relevant design. The model’s results turnaround time (2 hours) reduces early screening report time by 80% compared with endoscopy [5], making it a practical auxiliary tool for high-risk population screening.

Calibration curves (deviation <5% in 0.2–0.8 threshold range) and DCA confirm the model’s clinical utility. For high-risk individuals (predicted probability >0.3), further endoscopic examination can improve early diagnosis rates; for low-risk individuals (predicted probability <0.1), unnecessary invasive procedures are avoided, potentially reducing unnecessary endoscopic procedures and associated medical costs, while alleviating patient anxiety.

SHAP analysis enhanced the model’s interpretability, with HCT, CST4, and Age as top contributors—findings with clear biological plausibility. HCT’s prominent role is consistent with Kiebach et al. [14], who reported low hematocrit associated with higher colorectal cancer prevalence and cancer-related fatigue due to tumor-induced chronic anemia—a condition that is not observed in most benign gastrointestinal diseases. Age is an established non-modifiable risk factor for gastrointestinal tumors [3], with incidence increasing significantly with age, while benign gastrointestinal diseases can occur in all age groups. CST4’s role in tumor progression (extracellular matrix hydrolysis and invasion [11]) is a specific malignant characteristic, with no significant elevation in benign lesions. These findings further validate the model’s biological rationality, supporting further clinical investigation.

This study has several limitations. First, in accordance with TRIPOD-AI guidelines, we acknowledge that the absence of external validation is a critical methodological gap: while internal validation (5-fold cross-validation, bootstrap, calibration, and learning curves) provided optimism-corrected estimates, it cannot establish model transportability across different centers or patient spectra. Second, the single-center retrospective design and case-control sampling may inflate diagnostic performance compared with real-world screening populations. Third, the model is suitable for high-risk targeted screening but not general population mass screening. Finally, only laboratory indicators were incorporated; future studies should integrate clinical and imaging data and prioritize prospective multi-site external validation before any clinical deployment.

## Conclusion

This internally validated non-invasive tool demonstrates promising preliminary performance for early screening of gastrointestinal tumors in high-risk populations, with a high sensitivity of 95.4% and modest specificity of 61.5%. Owing to its relatively modest specificity, the model is not suitable for large-scale general population screening, but its use of routinely accessible laboratory indicators makes it a potentially useful auxiliary tool for early risk stratification in high-risk individuals. Further external prospective multi-center validation is mandatory before any clinical application.

## Supporting information

S1 Table Baseline characteristics and comparison between gastrointestinal tumor patients and controls. (PDF)

S2 Table Projected Positive and Negative Predictive Values at Different Disease Prevalence Levels. (PDF)

S3 Table Sensitivity, stratified, and age/sex-matched analyses of the SVM model. (PDF)

S4 Table Hyperparameter Search Grids for the 11 Machine Learning Classifiers. (PDF)

S1 Fig Learning curve analysis for sample size evaluation. (TIF)

S2 Fig The machine learning analysis pipeline. (TIF)
