# Supplementary material for: Serum Cystatin 4 combined with routine clinical laboratory indicators: A support vector machine diagnostic model for early screening of gastrointestinal tumors
Source: PLoS One. 2026 Sep 21;21(9):e0353753. doi: 10.1371/journal.pone.0353753 (PMC13592712; doi:10.1371/journal.pone.0353753)
Supplement: Supplementary file 1 [file pone.0353753.s001.pdf]

Table S1: Baseline characteristics and comparison between gastrointestinal tumor patients and controls

Characteristic	Subgroup	Gastrointestinal Tumors (n=214)	Control (n=130)	*P* value
Age, years	Mean ± SD	62.1 ± 10.0	50.9 ± 13.3	$< 0.001^{a}$
	Range	32–81	40–88	—
Sex, n (%)	Male	144 (67.3)	66 (50.8)	0.003^{b}
	Female	70 (32.7)	64 (49.2)	—
Histological subtype, n (%)	Gastric cancer	91 (42.5)	—	—
	Colorectal cancer	80 (37.4)	—	—
	Esophageal cancer	43 (20.1)	—	—
TNM stage/T1-TX, n (%)	T1	9 (4.2)	—	—
	T2	32 (15.0)	—	—
	T3	142 (66.4)	—	—
	T4	30 (14.0)	—	—
	TX	1 (0.5)	—	—
TNM stage/N0-NX, n (%)	N0	64 (29.9)	—	—
	N1	54 (25.2)	—	—
	N2	55 (25.7)	—	—
	N3	36 (16.8)	—	—
	NX	5 (2.3)	—	—
TNM stage/M0-MX, n (%)	M0	169 (79.0)	—	—
	M1	36 (16.8)	—	—
	MX	9 (4.2)	—	—
Tumor stage, n (%)	Stage I	14 (6.5)	—	—
	Stage II	59 (27.6)	—	—
	Stage III	92 (43.0)	—	—
	Stage IV	49 (22.9)	—	—

Note: [a] Independent samples *t*-test; [b] Pearson chi-square test. TNM = tumor-node-metastasis; SD = standard deviation.
