# Supplementary material for: Serum Cystatin 4 combined with routine clinical laboratory indicators: A support vector machine diagnostic model for early screening of gastrointestinal tumors
Source: PLoS One. 2026 Sep 21;21(9):e0353753. doi: 10.1371/journal.pone.0353753 (PMC13592712; doi:10.1371/journal.pone.0353753)
Supplement: Supplementary file 2 [file pone.0353753.s002.pdf]

Table S2: Projected Positive and Negative Predictive Values at Different Disease Prevalence Levels

**Assumed Prevalence (P)**	**Clinical Scenario**	**Positive Predictive Value (PPV)**	**Negative Predictive Value (NPV)**
62.2%	Current study (case-control test set)	80.5% (observed)	88.9% (observed)
15%	High-risk clinic	∼29.8%	∼99.2%
10%	High-risk clinic	∼21.4%	∼99.2%
5%	High-risk clinic/ symptomatic cohort	∼11.5%	∼99.6%
1%	High-risk community screening	∼2.4%	∼99.9%
0.1%	General population screening	∼0.25%	∼99.99%

Notes: [a] Values for prevalence = 62.2% are the observed performance of the SVM model in the independent test set ($n = 104$).
[b] Values for other prevalence levels are theoretical projections calculated using Bayes' theorem with the following formulas: $\text{PPV} = (\text{Se} \times P)/[\text{Se} \times P + (1 - \text{Sp}) \times (1 - P)]$; $\text{NPV} = (\text{Sp} \times (1 - P))/[\text{Sp} \times (1 - P) + (1 - \text{Se}) \times P]$, where $\text{Se} = 0.954$, $\text{Sp} = 0.615$, and P = assumed prevalence.
[c] The dramatic decrease in PPV with decreasing prevalence highlights the critical limitation of this case-control study: the high disease prevalence artificially inflates PPV, and the model's performance in a real-world screening setting with low prevalence is expected to be substantially worse.
