# Supplementary material for: Serum Cystatin 4 combined with routine clinical laboratory indicators: A support vector machine diagnostic model for early screening of gastrointestinal tumors
Source: PLoS One. 2026 Sep 21;21(9):e0353753. doi: 10.1371/journal.pone.0353753 (PMC13592712; doi:10.1371/journal.pone.0353753)
Supplement: Supplementary file 3 [file pone.0353753.s003.pdf]

Table S3: Sensitivity, stratified, and age/sex-matched analyses of the SVM model

**Analysis**	**Subgroup / Condition**	n	**AUC (95% CI)**	**Interpretation**
Original model	Full testing set	104	0.85 (0.77–0.92)	Baseline performance
Matched model	Age/sex-matched training<br>Full testing	132 (66 pairs)	0.84 (0.76–0.91)	Age-balanced retraining
Stratified	<55 years	36	0.89 (0.76–1.00)	Robust discrimination
	55–65 years	31	0.86 (0.72–0.99)	Robust discrimination
	>65 years	37	0.58 (0.36–0.80)	Attenuated in elderly
Sensitivity	Excluding age (6 features)	104	0.86 (0.78–0.93)	Age not primary driver

Notes: [a] The original model represents the baseline SVM performance on the full independent test set ($n = 104$). [b] The matched model was retrained using age- and sex-matched case-control pairs (66 pairs; $n = 132$ total), and evaluated on the full test set to assess whether age/sex imbalance affected baseline performance. [c] Stratified analyses by age group were performed in the full test set to evaluate subgroup-specific discrimination. [d] The sensitivity analysis excluded the age variable from the model, retaining the remaining 6 features, to assess whether age was the primary driver of model performance.
AUC = area under the receiver operating characteristic curve; CI = confidence interval.
