# Supplementary material for: Serum Cystatin 4 combined with routine clinical laboratory indicators: A support vector machine diagnostic model for early screening of gastrointestinal tumors
Source: PLoS One. 2026 Sep 21;21(9):e0353753. doi: 10.1371/journal.pone.0353753 (PMC13592712; doi:10.1371/journal.pone.0353753)
Supplement: Supplementary file 4 [file pone.0353753.s004.pdf]

Table S4: Hyperparameter Search Grids for the 11 Machine Learning Classifiers

**Classifier**	**Parameter**	**Search Range**
Naïve Bayes	alpha	[0.1, 1.0, 10.0]
k-Nearest Neighbors	n_neighbors	[1, 3, 4, 7, 8, 10]
	p	[1, 3, 5, 6]
Logistic Regression	C	[0.001, 0.009, 0.01, 0.09, 1, 5, 10, 25]
	penalty	['l1', 'l2']
	solver	['liblinear', 'saga']
Random Forest	n_estimators	[10, 100, 200]
	max_depth	[3, 5, 10, None]
	max_features	[3, 5, 7]
Decision Tree	max_depth	[1, 2, ..., 19]
	criterion	['gini', 'entropy']
Artificial Neural Network	hidden_layer_sizes	[(5, 5), (100, 100), (200, 200)]
	max_iter	[500, 1000, 1500]
Support Vector Machine	C	[0.1, 1, 10, 100]
	kernel	['linear', 'rbf']
Gradient Boosting	n_estimators	[100, 150, 200, 250, 300]
AdaBoost	n_estimators	[50, 100, 200]
	learning_rate	[0.1, 0.95, 1.0]
XGBoost	n_estimators	[80, 84, 88, ..., 196]
LightGBM	num_leaves	[31, 50, 100]
	max_depth	[3, 5, -1]
	learning_rate	[0.01, 0.05, 0.1]

Notes: All hyperparameters were optimized using GridSearchCV with stratified 5-fold cross-validation, maximizing AUC-ROC. Parameters not listed here (e.g., weights, algorithm, activation, gamma) were fixed at their default values and not included in the search grid. $\text{range}(1, 20) = [1, 2, \ldots, 19]$; None = no explicit limit. For SVM, the optimal kernel was consistently linear across all folds.
