# Supplementary material for: Serum Cystatin 4 combined with routine clinical laboratory indicators: A support vector machine diagnostic model for early screening of gastrointestinal tumors
Source: PLoS One. 2026 Sep 21;21(9):e0353753. doi: 10.1371/journal.pone.0353753 (PMC13592712; doi:10.1371/journal.pone.0353753)
Supplement: Supplementary file 5 [file pone.0353753.s007.pdf]

Reviewer Comments

By Chunzi Liang

This manuscript addresses an important topic and applies multiple machine-learning models; however, substantial revisions are required before the work can be adequately evaluated.

1. **Language and figure/table quality control**
   There are multiple instances of Chinese text appearing in figures/tables in an English-language manuscript. This is a basic but critical presentation issue. Please ensure that all figure labels, legends, axis titles, footnotes, and table content are fully in English and professionally proofread throughout.
2. **Feature-engineering results need clearer visualization and methodological transparency**
   The current presentation of feature-engineering outputs (e.g., check-mark tables across multiple models) is difficult to interpret and does not clearly communicate the rationale for feature optimization/selection.
   - Please replace or complement the current tables with more informative visual summaries (e.g., overlap/upset-style plots, ranked importance plots, or model-wise stability summaries).
   - Clearly define how "common features" were identified across models, including thresholds/criteria, and explain whether this step represents feature selection, feature reduction, or post hoc interpretability analysis.
3. **Statistical analysis concerns (including one-way p-values)**
   The use and placement of one-way p-values in the current section appear methodologically inappropriate or at least insufficiently justified.
   - Please clarify the statistical objective of these tests and why they are relevant to the machine-learning pipeline at that stage.
   - Reassess whether univariate significance testing is being over-interpreted for multivariable predictive modeling.

    - Improve data reporting consistency (test names, assumptions, exact p-value formatting, multiple-testing handling where applicable, and coherent alignment between text, tables, and figures).

4. **Validation strategy is insufficient for claims of robustness/generalizability**
   The study lacks external validation and does not include prospective comparison. This substantially limits evidence for model transportability and real-world utility.
    - At minimum, include an external validation cohort (from a different site/time period/population) or clearly down-scope conclusions to internal performance only.
    - If prospective validation is not currently feasible, explicitly acknowledge this as a major limitation and provide a concrete future validation plan.

5. **Recommendation**
   Given the above issues, I recommend **major revision**. The manuscript may become suitable for reconsideration after substantial improvements in reporting quality, feature-engineering presentation, statistical rigor, and validation design.
