# Supplementary material for: Serum Cystatin 4 combined with routine clinical laboratory indicators: A support vector machine diagnostic model for early screening of gastrointestinal tumors
Source: PLoS One. 2026 Sep 21;21(9):e0353753. doi: 10.1371/journal.pone.0353753 (PMC13592712; doi:10.1371/journal.pone.0353753)
Supplement: Supplementary file 6 [file pone.0353753.s009.docx]

**Response to Reviewers**

**Manuscript ID**: PONE-D-26-02453

**Title**: **Serum Cystatin 4 Combined with Routine Clinical Laboratory Indicators: A Support Vector Machine Diagnostic Model for Early Screening of Gastrointestinal Tumors**

We sincerely thank the academic editor and two reviewers for their constructive and insightful comments, which have significantly improved the quality and rigor of our manuscript. We have carefully addressed all raised issues with detailed revisions and supplementary analyses, and the major modifications to the manuscript are highlighted with track changes. Below is a point-by-point response to each comment.

**Responses to Reviewer #1 (Chunzi Liang)**

1. Language and figure/table quality control

**Comment**: Multiple Chinese text in figures/tables; need full English proofreading for all labels, legends, axis titles, footnotes and table content.

**Response**: We have fully revised all figures and tables in the manuscript: (1) All Chinese text in figure labels, legends, axis titles, and table footnotes has been replaced with standard English; (2) The entire manuscript (including all figures, tables and supplementary materials) has been professionally proofread for English expression, grammar and typography; (3) We have standardized the naming and formatting of all figures/tables to meet PLOS ONE’s publication requirements. All revised figures and tables are provided in the main manuscript and supporting information.

2. Feature-engineering results need clearer visualization and methodological transparency

**Comment**: Current check-mark tables are hard to interpret; need informative visual summaries (overlap/upset plots, ranked importance plots); define "common features" identification criteria and clarify feature selection/reduction/interpretability analysis.

**Response**: We have comprehensively optimized the presentation and methodological description of feature engineering:

(1) Visualization improvement: Replaced the original check-mark tables with algorithm-specific feature importance ranked plots (Fig 3A-D) and an upset-style overlap plot (Fig 4) to clearly show the feature selection results across four algorithms (random forest, decision tree, LASSO regression, RFE). These plots intuitively display the ranking of all 38 variables and the overlapping core features identified by multiple algorithms.

(2) Methodological definition: We have clearly defined the criteria for identifying "common features" in the Materials & Methods - Feature Selection section: features identified by all four algorithms were prioritized; features with high ranking and high co-occurrence frequency across methods were preferentially selected; features with high model importance and clear clinical significance were additionally incorporated. We explicitly state that this step is a feature selection process (combining consensus selection and multicollinearity elimination) performed exclusively on the training set to avoid data leakage, with post hoc interpretability analysis completed via SHAP analysis in the results section.

(3) Supplementary details: We added a Pearson correlation heatmap (Fig 5) to show the multicollinearity between candidate features, and clearly explain the elimination of HGB (r=0.94 with HCT) to retain non-redundant core variables.

3. Statistical analysis concerns (including one-way p-values)

**Comment**: Inappropriate use/placement of one-way p-values; clarify statistical objective and relevance to ML pipeline; reassess univariate significance interpretation; improve data reporting consistency.

**Response**: We have revised the statistical analysis section and standardized p-value reporting with the following key changes:

(1) Clarify statistical objective: We explicitly state in the Statistical Analysis section that univariate comparison p-values are purely descriptive for baseline between-group differences of indicators, and are not used for variable selection in the machine learning pipeline (all feature selection relies on four dedicated ML algorithms). This addresses the relevance of univariate tests to the overall study design (baseline characteristic description) rather than predictive modeling.

(2) Avoid over-interpretation: We removed any potential over-interpretation of univariate p-values in the original manuscript and emphasize that multivariable predictive modeling is the core of the study, with univariate results only serving as preliminary descriptive analysis.

4. Validation strategy is insufficient for claims of robustness/generalizability

**Comment**: Lack of external validation and prospective comparison; need external validation cohort or down-scope conclusions; acknowledge limitation and provide future validation plan if prospective is not feasible.

**Response**: We have revised the manuscript to address the validation limitations and provide a concrete future plan, with key changes:

(1) Down-scope conclusions: We have adjusted all conclusions in the abstract, discussion and conclusion sections to focus on internal performance of the model, and explicitly state that the model is currently suitable for targeted screening in gastrointestinal tumor high-risk populations (rather than general population mass screening) due to the lack of external validation.

(2) Acknowledge major limitation: We added a dedicated paragraph in the Discussion - Limitations section to highlight that the single-center retrospective design and lack of external prospective validation are the major limitations of the study, which may restrict the generalizability of the model.

5. Overall recommendation (Major revision)

**Response**: We have addressed all the four major concerns raised by Reviewer #1 with substantial revisions, including full English optimization of figures/tables, improved feature engineering visualization and methodological transparency, standardized statistical analysis and p-value reporting, and revised validation strategy with a concrete future plan. All revisions are highlighted in the tracked manuscript, and supplementary figures/tables are added to support the changes.

**Responses to Reviewer #2 (Pritam Goswami)**

**Major Comments**

1. Mismatch between title and Introduction section

**Comment**: Title focuses on CST4 + routine indicators SVM model, but Introduction emphasizes general GI malignancy burden without clear lead-in to biomarker-based screening; revise Introduction or modify title.

**Response**: We have comprehensively revised the Introduction section (instead of modifying the title) to align with the study’s core focus, with key revisions:

(1) We added a dedicated paragraph to highlight the limitations of current serum tumor markers (high false-positive/negative rates, poor performance in differentiating tumors from benign lesions) and the rationale for integrating novel biomarkers (CST4) with routine laboratory indicators (routine indicators are widely accessible, reflect systemic tumor responses, and complement specific biomarkers).

(2) We strengthened the connection between the study background (GI tumor screening dilemma) and the research objective (constructing a CST4-based multi-indicator ML model), and clearly state the innovation of the study: integrating a novel biomarker with routinely detectable laboratory indicators using multiple ML algorithms for early screening of GI tumors.

(3) We trimmed the overly general content about GI malignancy burden and prognosis, and focused on the unmet clinical need for non-invasive, accessible screening tools—the core motivation of the study.

2. Retrospective case-control design with spectrum bias; controls lack subclinical GI pathology screening; need prospective validation

**Comment**: Cancer vs. healthy/benign control design may inflate performance; clarify control screening status; discuss spectrum bias; recommend prospective screening cohort validation.

**Response**: We have addressed this issue with detailed explanations and revisions:

(1) Clarify control screening status: We added explicit information in the Materials & Methods - Research Subjects section that all control subjects (healthy individuals and benign GI disease patients) underwent imaging and laboratory examinations to rule out subclinical malignant lesions, with no history of malignant tumors, severe chronic inflammatory disorders or organ insufficiency.

(2) Explicitly discuss spectrum bias: We added a detailed discussion of spectrum bias in the Discussion - Limitations section, acknowledging that the case-control design (with relatively well-characterized controls) may inflate the diagnostic performance of the model compared with real-world screening scenarios, and that the model’s performance needs to be verified in unselected populations.

(3) Strengthen prospective validation recommendation: We included a prospective screening study in the concrete future validation plan (as stated in the response to Reviewer #1), and emphasize that the prospective study will use an unselected high-risk population to reduce spectrum bias and improve real-world applicability.

3. No sample size/power calculation; small dataset (344 participants) + 38 predictors risk model instability/overfitting

**Comment**: Need formal justification of sample size relative to predictors; address overfitting risk for ML modeling.

**Response**: We have added a formal sample size justification in the Materials & Methods - Research Subjects section and taken multiple measures to reduce overfitting risk:

(1) Sample size justification: We used the events-per-variable (EPV) criterion (the gold standard for diagnostic ML modeling sample size evaluation) to justify the sample size: (a) Initial EPV (214 cases/38 predictors) = 5.63, meeting the minimum acceptable EPV of 5; (b) After feature selection (7 core variables), EPV increased to 30.57, well above the recommended EPV of 10 for robust model construction. This effectively reduces the risk of model instability and overfitting.

(2) Overfitting mitigation measures: We added details of multiple strategies to avoid overfitting in the Materials & Methods - Algorithm Selection and Model Training section: (a) Exclusive feature selection on the training set to avoid data leakage; (b) 70/30 random dataset splitting into training/test sets; (c) 5-fold cross-validation for hyperparameter optimization; (d) Feature reduction from 38 to 7 non-redundant core variables; (e) Standardized hyperparameter tuning for all 11 ML algorithms.

(3) Supplementary evidence: We added the calibration curve of the SVM model (Fig 7/9) with a deviation <5% in the 0.2-0.8 threshold range, which confirms the model has no significant overfitting (good consistency between predicted and actual probabilities).

4. Unsubstantiated claims of "large-scale screening" and cost reduction; no cost-effectiveness analysis; low sensitivity (80.3% in original) + no PPV reported

**Comment**: Temper large-scale screening claims; provide PPV/NPV at plausible prevalence; address cost-effectiveness and sensitivity issues.

**Response**: We have fully revised the relevant claims and added all missing performance indicators, with key changes:

(1) Temper screening claims: We removed all claims of "large-scale screening" in the manuscript and revised to state that the model is suitable for targeted screening in high-risk populations (age >40 years, family history, chronic GI diseases) due to relatively low specificity and lack of external validation. We explicitly state in the abstract and conclusion that the model is not suitable for general population mass screening.

(2) Add PPV/NPV estimates: We calculated and reported PPV and NPV at different plausible prevalence rates (5%, 10%, 15% for high-risk populations; 62.2% for the study cohort) using the Bayesian method, and presented the results in Supplementary Table S2 (Projected Positive and Negative Predictive Values at Different Disease Prevalence Levels). We also report the test set PPV (80.5%) and NPV (88.9%) in the abstract, results and conclusion sections.

(3) Correct sensitivity value and explanation: The original 80.3% sensitivity was a typo; the correct test set sensitivity of the SVM model is 95.4% (we have corrected this typo throughout the manuscript). We explain that the high sensitivity (95.4%) makes the model an excellent rule-out test for high-risk populations, which is the key clinical value of the model (minimizing missed diagnoses).

(4) Address cost-effectiveness and cost reduction: We added a qualitative analysis of cost reduction in the Discussion section (avoiding unnecessary endoscopy reduces medical costs by ~300-500 RMB per person based on average Chinese endoscopy fees) and acknowledge that a formal cost-effectiveness analysis is lacking (added as a limitation). We plan to include cost-effectiveness analysis in the future prospective study.

Minor Comments

1. Inconsistencies in software versions (Python 3.10 vs 3.9)

**Response**: We have standardized all software versions in the Materials & Methods - Software and Tools section: the entire study used Python 3.10 for data processing, model construction and visualization (the original 3.9 was a typo, and we have corrected this throughout the manuscript). We also added the version numbers of all key libraries (scikit-learn 1.5.2, XGBoost 1.7.4, SHAP 1.4.6.1, R 4.5.2) for reproducibility.

2. Missing detail on missing data proportion and imputation impact

**Response**: We added detailed information on missing data in the Materials & Methods - Data Preprocessing section: (1) The overall missing data proportion across all 38 indicators and 344 subjects was 2.6%, with no single indicator having missing data >5%; (2) We used the random forest algorithm for missing value imputation, and added a validation result: the sensitivity/specificity change of the model after imputation was <0.5%, indicating that imputation had a negligible impact on the model performance.

3. Unspecified whether CST4 ELISA kits were validated for clinical diagnostic use

**Response**: We added explicit information in the Materials & Methods - Sample Collection and Indicator Testing section that the CST4 ELISA kit (Shanghai Liangrun Biomedical Technology Co., Ltd) has a certificate number: 20173403280 and meets CLSI quality control standards for quantitative in vitro diagnostic tests, confirming its validation for clinical diagnostic use.

4. Improve clarity regarding model hyperparameter tuning

**Response**: We added detailed information on hyperparameter tuning in the Materials & Methods - Algorithm Selection and Model Training section: (1) Hyperparameters for all 11 ML algorithms were optimized using grid search with 5-fold cross-validation on the training set; (2) We specified the key hyperparameters of the optimal SVM model (radial basis function kernel, regularization parameter C=1.0); (3) We state that the grid search range for each algorithm was set based on standard ML practice for diagnostic modeling, and the optimal hyperparameters were selected based on cross-validation AUC.

5. Language editing for Discussion fluency

**Response**: The entire Discussion section has been professionally edited for English fluency and logical flow: we reorganized the paragraphs to strengthen the connection between results and discussion, trimmed redundant content, and standardized the expression of clinical and statistical terms. We also used ChatGPT 3.5 to assist in refining the logical flow (with full review and validation by the authors, as stated in the Declaration of AI Assistance section).

Overall Comment

**Response**: We sincerely thank Reviewer #2 for the positive evaluation of our study (clinically relevant topic, strong interpretability with calibration curves/SHAP/DCA) and constructive comments. We have addressed all major and minor comments with detailed revisions, corrected all typos and inconsistencies, and added supplementary information/analyses to improve the rigor and reproducibility of the study. All revisions are highlighted in the tracked manuscript.
