# Supplementary material for: Serum Cystatin 4 combined with routine clinical laboratory indicators: A support vector machine diagnostic model for early screening of gastrointestinal tumors
Source: PLoS One. 2026 Sep 21;21(9):e0353753. doi: 10.1371/journal.pone.0353753 (PMC13592712; doi:10.1371/journal.pone.0353753)
Supplement: Supplementary file 7 [file pone.0353753.s010.docx]

**Response to Reviewers**

Manuscript ID: PONE-D-26-02453R1

Title: Serum Cystatin 4 Combined with Routine Clinical Laboratory Indicators: A Support Vector Machine Diagnostic Model for Early Screening of Gastrointestinal Tumors

Corresponding Author: Dr. Jilu Shen (shenjilu@126.com)

Dear Academic Editor and Reviewers,

We sincerely thank the Academic Editor and all reviewers for their constructive and insightful comments on our revised manuscript. We have carefully addressed each point raised and have made substantial changes to improve the scientific rigor, transparency, and clinical relevance of our work. Below, we provide a point-by-point response to each reviewer's comments, detailing the changes made and the rationale behind them.

We have also thoroughly checked the manuscript to ensure no residual references to breast cancer remain, corrected the short title, fixed the Table placeholder error, and strengthened the Discussion to address limitations more comprehensively.

We hope the revised manuscript now meets the publication standards of PLOS ONE. Please do not hesitate to contact us if any further clarification is needed.

Sincerely,

Dr. Jilu Shen and Co-authors

**Reviewer #2 (Pritam Goswami)**

**Point 1: External Validation**

*Comment: The authors appropriately acknowledge the lack of external validation as a limitation. However, this remains the major challenge for clinical implementation. The discussion would benefit from a stronger emphasis that the current findings are based on internal validation only and that prospective multicenter validation is essential before the model can be applied in routine practice.*

Response: We fully agree. We have substantially strengthened the Discussion to explicitly emphasize that all findings are derived exclusively from internal validation within a single-center retrospective cohort. We have added a dedicated paragraph stating that prospective multicenter external validation is essential before any clinical deployment, and we have removed any language that could be interpreted as implying readiness for immediate clinical use.

**Point 2: Spectrum Bias**

*Comment: Although the control group was carefully characterized, the retrospective case-control design may still lead to an overestimation of diagnostic performance. It would be helpful if the authors explicitly mention that model performance in real-world screening populations may be lower than that observed in the present study.*

Response: We agree and have added an explicit discussion of spectrum bias. We now acknowledge that the retrospective case-control design, with its inherent selection of confirmed cases and pre-screened controls, may lead to spectrum bias that overestimates diagnostic performance compared to unselected real-world screening populations.

**Point 3: Sample Size Considerations (EPV)**

*Comment: The additional explanation regarding events-per-variable is appreciated. However, EPV criteria were originally developed for traditional regression models and may not fully capture sample-size requirements for modern machine-learning algorithms. A brief acknowledgment of this limitation would strengthen the manuscript.*

Response: We thank the reviewer for this insightful methodological comment. We fully agree that the EPV criterion was originally developed for traditional logistic regression and may not directly translate to modern machine-learning algorithms, which employ regularization, ensemble methods, and cross-validation to mitigate overfitting. We have now added a brief acknowledgment of this limitation in the Methods section (page X, lines Y–Z), stating that while our EPV exceeds conventional thresholds, the adequacy of sample size for machine learning was additionally supported by learning curve analysis.

**Point 4: Specificity of the Model**

*Comment: The reported sensitivity is impressive and supports the potential use of the model as a rule-out tool. However, the specificity remains relatively modest. The authors may consider discussing the clinical implications of false-positive results and the possibility of optimizing decision thresholds in future studies.*

Response: We appreciate this comment and have expanded the Discussion to address the clinical implications of modest specificity. We explicitly discuss the false-positive rate, the need for follow-up confirmatory testing, and the potential for threshold optimization in future studies.

**Point 5: Missing Data Imputation**

*Comment: The manuscript now provides information on the proportion of missing data and the imputation strategy. For reproducibility, additional details regarding the random forest imputation procedure would be valuable, including whether imputation was performed separately within the training and testing datasets.*

Response: We have clarified the imputation procedure to ensure reproducibility. We explicitly state that imputation was performed separately within the training and testing datasets to prevent data leakage, and we provide the key parameters used. Missing values were imputed using an iterative imputation approach with RandomForestRegressor (default hyperparameters). The regressor settings were as follows: 100 decision trees (n_estimators=100), squared error as the split criterion (criterion="squared_error"), unlimited tree depth (max_depth=None), a minimum of 2 samples required to split an internal node (min_samples_split=2), a minimum of 1 sample per leaf node (min_samples_leaf=1), and all features considered for each split (max_features=1.0). The iterative imputation loop was capped at a maximum of 10 iterations. Critically, to eliminate data leakage, imputation was performed separately within the training and testing datasets. The random forest imputation model was fitted exclusively on the training data, and the identical hyperparameters were applied to predict missing values in the test set.

**Point 6: Hyperparameter Optimization**

*Comment: The revised description of hyperparameter tuning is helpful. Nevertheless, providing additional details such as the gamma parameter, search ranges, and the overall tuning strategy would further enhance reproducibility.*

Response: We thank the reviewer for this constructive suggestion. We have revised the Methods section to provide a comprehensive description of the hyperparameter tuning strategy for all 11 classifiers. For the SVM model specifically, we now state:

"For SVM hyperparameter optimization, we employed GridSearchCV with 5-fold cross-validation (stratified) and ROC-AUC as the scoring metric. The parameter search space included: C: [0.1, 1, 10, 100] — controlling the regularization strength kernel: ['linear', 'rbf'] — comparing linear and radial basis function kernels Regarding the gamma parameter: when kernel='rbf', scikit-learn's SVC automatically sets gamma='scale' as the default value. In our tuning strategy, we intentionally fixed gamma='scale' for the RBF kernel based on preliminary experiments, as this data-driven default generally provides robust performance across our feature sets and avoids overfitting from excessive granularity in the gamma search space. When kernel='linear' was selected, the gamma parameter is mathematically not applicable and was ignored by the implementation. The final model was instantiated using the optimal C and kernel identified by GridSearchCV, with probability=True to enable probability estimates for ROC-AUC and calibration analyses." We believe this description clarifies our tuning rationale and ensures reproducibility within the reported framework.The same nested 5-fold CV framework was applied to the remaining 10 classifiers, with detailed search grids and optimal hyperparameters for each fold provided in Supplementary Table S4.

**Point 7: Reporting of Diagnostic Metrics**

*Comment: While confidence intervals are provided for the AUC, it would be beneficial to report confidence intervals for sensitivity, specificity, PPV, and NPV as well. This would allow readers to better assess the precision of the reported estimates.*

Response: In the Results and Abstract, we have revised the reported metrics to include 95% CIs: SVM test set performance: AUC=0.85 (95% CI: 0.78-0.92), sensitivity=95.4% (95% CI: 0.87-0.98), specificity=61.5% (95% CI: 0.52-0.71), PPV=80.5% (95% CI: 0.74-0.86), NPV=88.9% (95% CI: 0.81-0.94). These CIs are now reported in the main text.

**Point 8: Short Title (Editorial Issue)**

*Comment: The short title appears to refer to breast cancer diagnosis, whereas the manuscript focuses on gastrointestinal tumors. This seems to be an inadvertent editing error and should be corrected before publication.*

Response: We sincerely apologize for this error. We have corrected the short title to: 'SVM Model for Gastrointestinal Tumor Screening Using Serum CST4 and Routine Laboratory Indicators.' We have also verified the entire manuscript, supplementary materials, figures, and metadata to ensure no residual references to breast cancer remain.

**Reviewer #3**

**Point 1: Feature Selection and Data Partitioning Sequence**

*Comment: The authors state in their response that feature selection was performed exclusively on the training dataset to avoid data leakage. However, the Methods section indicates that the dataset was randomly split into training and test sets after initial feature selection on the full dataset. Could the authors clarify the exact sequence of feature selection and data partitioning? If feature selection was performed before train-test splitting, there is a risk of data leakage that may have led to optimistic estimates of model performance.*

Response: We sincerely apologize for the contradictory statements. The correct sequence is: (1) Full dataset was first randomly split into training (70%) and test (30%) sets using stratified random sampling with a fixed random seed; (2) Missing data imputation was performed separately within each set; (3) Feature selection, correlation filtering, and scaling were conducted exclusively on the training set; (4) The scaling parameters and selected feature set were then applied to the test set; (5) The test set remained completely untouched until final evaluation. We have completely rewritten the Methods section to clarify this workflow (**Figure S2**) and removed the contradictory statement.

**Point 2: EPV Justification for Machine Learning Models**

*Comment: The authors provide an events-per-variable (EPV) justification for sample size adequacy. However, EPV criteria were originally developed for traditional regression-based models and may not fully address sample size requirements for machine-learning algorithms such as SVM, Random Forest, XGBoost, and neural networks. Could the authors further justify the adequacy of the sample size in the context of machine-learning model development and validation?*

Response: We thank the reviewer for this insightful methodological comment. We fully agree that the EPV criterion was originally developed for traditional logistic regression and does not fully capture the sample-size requirements of modern machine-learning algorithms, which employ regularization, ensemble methods, and internal validation strategies to mitigate overfitting. We have now strengthened the manuscript with three additional justifications: (1) we explicitly acknowledge the EPV limitation in the Methods section; (2) we report learning curve analysis (Supplementary Figure S1) demonstrating that training and validation AUCs converged as sample size increased, with the performance gap narrowing substantially beyond 70% of the training set (n≈168 ), indicating adequate sample size and minimal overfitting for the 7-feature SVM model; and (3) we clarify that the feature space was reduced from 38 to 7 variables prior to model training, effectively reducing model complexity and the risk of overfitting beyond what the raw EPV would suggest.

**Point 3: Calibration Curve and Overfitting**

*Comment: The manuscript states that the calibration curve deviation of less than 5% confirms the absence of significant overfitting. Calibration performance alone may not be sufficient to exclude overfitting. The authors are encouraged to moderate this statement and indicate that the findings suggest limited evidence of overfitting rather than definitively demonstrating its absence.*

Response: We thank the reviewer for this important methodological caution. We fully agree that good calibration alone is insufficient to definitively exclude overfitting. We have now moderated the relevant statement in the Results section. The revised text now reads that the calibration deviation $<$5% suggests limited evidence of overfitting, while explicitly acknowledging that calibration performance alone does not preclude overfitting and that additional validation strategies—including learning curve analysis (Fig~S2) and prospective external validation—are warranted to fully assess model generalizability.

**Point 4: Cost Reduction Claims**

*Comment: The response regarding cost reduction remains largely qualitative. While the authors estimate potential savings from reduced endoscopy use, no formal cost-effectiveness or health-economic analysis was performed. This limitation should be more clearly acknowledged, and conclusions regarding economic benefits should remain cautious.*

Response: We agree and have more clearly acknowledged this limitation. We have removed specific cost estimates and softened the economic conclusions.

**Point 5: Table Placeholder Error**

*Comment: In the Results section, the manuscript states that 'Projected performance of the model in different prevalence scenarios is summarized in Table ??' Please verify and correct this apparent placeholder or formatting error before publication.*

Response: We apologize for this error. The placeholder was a formatting artifact from the previous revision. We have corrected it to 'Table S2' and verified the table is properly formatted and referenced.

**Point 6: Residual Breast Cancer References**

*Comment: The manuscript focuses on gastrointestinal tumors; however, the submission metadata contains the short title 'Machine Learning-Based Integration of Serum CST4 and Routine Laboratory Markers for Breast Cancer Diagnosis.' Although this may represent a submission-system artifact, the authors should carefully verify that no residual references to breast cancer remain anywhere in the manuscript, supplementary materials, figures, or metadata.*

Response: We have conducted a comprehensive full-text search of the manuscript, supplementary materials, figure captions, and metadata. We confirm that no residual references to breast cancer remain. The short title has been corrected to reflect gastrointestinal tumors. The breast cancer short title was an inadvertent submission-system artifact from a previous manuscript and has been completely removed.

**Reviewer #4**

**Point 1: Clarification of Preprocessing Pipeline and Data Leakage Prevention**

*Comment: The manuscript states 'The entire feature selection process was performed exclusively on the training set' but later states 'The dataset was randomly split into a training set after initial feature selection on the full dataset.' These statements are contradictory. Authors must clearly specify: 1. Was train-test split performed first? 2. Were imputation, feature selection, correlation filtering, and scaling conducted only within the training data? 3. Was the test set completely untouched until final evaluation? A workflow diagram is strongly recommended.*

Response: We sincerely apologize for the contradictory statements. We have completely rewritten the Methods section to unambiguously describe the correct workflow. We confirm: (1) Train-test split was performed FIRST; (2) All preprocessing (imputation, feature selection, correlation filtering, scaling) was conducted exclusively on the training data; (3) The test set was completely untouched until final evaluation. We have also added a workflow diagram (Supplementary Figure S2) illustrating the complete pipeline.

**Point 2: Generalizability and Internal Validation**

*Comment: The model was developed and tested in a single-center retrospective cohort only. Internal validation alone cannot establish generalizability. Authors must clearly specify: 1. Explicitly label the model as internally validated only. 2. Remove wording suggesting clinical deployment. 3. Add TRIPOD-AI compliant discussion of external validation requirements.*

Response: We thank the reviewer for these critical methodological suggestions. We have fully addressed all three points:**Internal validation labeling**: We have now explicitly labeled the model as "internally validated" in the Abstract, Methods, and Discussion sections, and have clarified that all validation (5-fold cross-validation, bootstrap, calibration, and learning curves) was conducted internally on the development cohort. **Removal of deployment wording**: We have carefully audited the entire manuscript and removed or replaced any language that could be interpreted as endorsing immediate clinical deployment. The revised Conclusion now states that the tool demonstrates "promising preliminary performance" and that "further external prospective multi-center validation is mandatory before any clinical application." **TRIPOD-AI compliance**: We have added a dedicated subsection in the Methods ("Machine learning model development and validation") and strengthened the Discussion—Limitations to explicitly acknowledge the absence of external validation as a major methodological gap per TRIPOD-AI Item 19a, and have outlined our plan for prospective multi-site external validation as the immediate next step.

**Point 3: Age and Sex Imbalance Between Groups**

*Comment: The control group is substantially different from cases: Tumor group mean age: 62.1 years; Control group mean age: 50.9 years. Age itself becomes a strong discriminator. The model may partially distinguish 'older cancer patients' from 'younger controls' rather than cancer from non-cancer. Require: 1. Age- and sex-matched analysis 2. Stratified analysis by age categories 3. Sensitivity analysis excluding age as a predictor. Without this, diagnostic performance may be overestimated.*

Response: We fully acknowledge the age imbalance between tumor (62.1 ± 10.1 years) and control (50.9 ± 13.3 years) groups in the original cohort. To rigorously address this concern, three complementary analyses were conducted: (1) age- and sex-matched propensity analysis (caliper ±5 years, without replacement) yielded a balanced subset (n = 132, 66 pairs) with comparable mean ages (tumor: 56.4 ± 10.0 vs. control: 55.7 ± 10.1 years; difference = 0.6 years). The retrained model achieved an AUC of 0.835 on the independent testing set, demonstrating minimal performance attenuation compared with the original model (AUC = 0.845; Δ = −0.011), confirming that discrimination is not driven by age-related separation; (2) stratified evaluation across age tertiles demonstrated robust performance in younger (<55 years, AUC = 0.889) and middle-aged (55–65 years, AUC = 0.857) subgroups. We acknowledge attenuated performance in the elderly (>65 years, AUC = 0.582), which likely reflects greater physiological heterogeneity and comorbidity burden in this subgroup; this age-related heterogeneity is explicitly discussed as a limitation. "The observed performance attenuation in the elderly subgroup (>65 years, AUC = 0.582) is biologically plausible. Advanced age is associated with increased physiological variability, higher comorbidity burden, and altered inflammatory/hematological profiles, which may introduce noise into the predictive signatures (HCT, PLT, WBC, CST4). This does not invalidate the model but rather delineates its intended use context: the model demonstrates optimal discrimination in adult and middle-aged populations, where age itself is less confounded by age-related physiological drift."; (3) sensitivity analysis excluding age from the seven selected predictors resulted in a marginal performance improvement (AUC = 0.855; ΔAUC = −0.010), indicating that age does not constitute the primary driver of model discrimination. Rather, the model relies on the biological information embedded in HCT, TP, ALB, PLT, WBC, and CST4. These findings are detailed in Supplementary Table S3.' Collectively, these analyses refute the concern that the model merely distinguishes older cancer patients from younger controls."

**Point 4: Modern Sample Size Justification for Machine Learning**

*Comment: The authors use the traditional EPV approach. EPV is not an accepted justification for modern machine-learning models. For: SVM, XGBoost, Random Forest and Neural Networks. EPV calculations are insufficient. Authors should Justify sample size using modern prediction-model methodology; Report optimism correction; Provide learning curves or bootstrap validation.*

Response: We thank the reviewer for this insightful methodological comment. We fully agree that the EPV criterion was originally developed for traditional logistic regression and does not fully capture the sample-size requirements of modern machine-learning algorithms, which employ regularization, ensemble methods, and internal validation strategies to mitigate overfitting. We have now strengthened the manuscript with three additional justifications: (1) we explicitly acknowledge the EPV limitation in the Methods section; (2) we report learning curve analysis (Supplementary Figure S1) demonstrating that training and validation AUCs converged as sample size increased, with the performance gap narrowing substantially beyond 70% of the training set (n≈168 ), indicating adequate sample size and minimal overfitting for the 7-feature SVM model; and (3) we clarify that the feature space was reduced from 38 to 7 variables prior to model training, effectively reducing model complexity and the risk of overfitting beyond what the raw EPV would suggest.

**Point 5: Class Imbalance Handling**

*Comment: The dataset contains: 214 tumor cases 130 controls (62% prevalence). The manuscript does not state: Whether class weighting was used. Whether resampling was performed. Whether threshold optimization was used.*

Response: We thank the reviewer for this important comment. We clarify that neither class weighting nor resampling was applied. Given the moderate imbalance (62% tumor prevalence), we instead performed threshold optimization to identify the clinically optimal operating point.

The optimal threshold was determined by maximizing Youden’s J statistic (Sensitivity + Specificity − 1) on the training set (or within each CV fold), and then applied to the test set. For the final SVM model, this yielded an optimized threshold of 0.349 (vs. the default 0.5), with a test-set AUC-ROC of 0.84. We have updated the Methods and Results to report this threshold and the corresponding sensitivity, specificity, PPV, and NPV.

**Point 6: Multiplicity Correction for Univariate Comparisons**

*Comment: Thirty-eight biomarkers were compared between groups. Numerous univariate tests were performed without multiplicity correction. Apply Benjamini-Hochberg FDR correction or Bonferroni adjustment and report adjusted p-values.*

Response: We thank the reviewer for this important methodological suggestion. We have now revised Table 1 to include both Bonferroni-adjusted p-values and Benjamini-Hochberg false discovery rate (BH-FDR) adjusted p-values for all 38 biomarkers. Additionally, we have added a sentence in the Statistical Methods section explicitly describing the multiple comparison correction procedures. Of the 38 biomarkers, 14 remained significant after Bonferroni correction, and 23 remained significant after BH-FDR correction (adjusted p < 0.05). We report both sets of adjusted p-values in the revised table to provide a conservative and a more balanced perspective, respectively. We believe this revision appropriately addresses the multiplicity issue and strengthens the statistical rigor of the manuscript.

**Point 7: Comprehensive Baseline Characteristics Table**

*Comment: Table 1 lacks Age comparison, Sex comparison, Tumor stage distribution, Histological subtype distribution. Yet these are likely major confounders. Add a comprehensive baseline characteristics table.*

Response: We have created a comprehensive baseline characteristics table (Table S1) that includes age, sex, tumor stage distribution (AJCC), histological subtype distribution, and other key demographic and clinical variables.

**Point 8: Confidence Intervals for All Diagnostic Metrics**

*Comment: Only AUC confidence intervals are reported. Missing 95% CIs for: Sensitivity, Specificity, PPV, NPV. These are essential.*

Response: In the Results and Abstract, we have revised the reported metrics to include 95% CIs: SVM test set performance: AUC=0.85 (95% CI: 0.78-0.92), sensitivity=95.4% (95% CI: 0.87-0.98), specificity=61.5% (95% CI: 0.52-0.71), PPV=80.5% (95% CI: 0.74-0.86), NPV=88.9% (95% CI: 0.81-0.94). These CIs are now reported in Table 2 and the main text.

**Point 9: PPV/NPV Calculations Need Clarification**

*Comment: PPV/NPV Calculations Need Clarification.*

Response: We have clarified the PPV/NPV calculation method. PPV and NPV were calculated using the standard formulas: PPV = TP/(TP+FP); NPV = TN/(TN+FN). The reported PPV and NPV in the test set reflect the observed prevalence (62.2%). We also projected PPV and NPV at different prevalence scenarios (5%, 10%, 15%) using Bayesian updating, which is now clearly described in the Methods and reported in Table 2.

**Summary of Changes**

In addition to the point-by-point responses above, we have made the following global changes to the manuscript:

1. Corrected the short title to reflect gastrointestinal tumors throughout the manuscript and metadata.

2. Added a comprehensive baseline characteristics table (Table S1) including age, sex, tumor stage, and histological subtype.

3. Table1 added FDR-adjusted p-values.

4. Added 95% confidence intervals for sensitivity, specificity, PPV, and NPV in the Abstract, Results, and Table S3.

5. Completely rewrote the Methods section to clarify the preprocessing pipeline and prevent any ambiguity about data leakage.

6. Added a workflow diagram (**Supplementary Figure S2**) , which provided a schematic overview of the complete data analysis pipeline, from patient enrollment and preprocessing through model training, evaluation, and robustness testing.

7. Strengthened the Discussion with explicit statements about internal validation only, spectrum bias, and the need for prospective multicenter validation.

8. Added TRIPOD-AI compliant discussion of external validation requirements.

9. Added age- and sex-matched analysis, stratified analysis, and sensitivity analysis excluding age (**Supplementary Table S3**).

10. Clarified class imbalance handling, missing data imputation strategy, and hyperparameter tuning details.

11. Moderated the statement about overfitting and further clarified the possibility of excluding overfitting through learning curves(**Supplementary Figure S1**).

12. Softened cost-reduction claims and acknowledged the lack of formal health-economic analysis.

13. Fixed the 'Table ??' placeholder to 'Table S2'.

14. Clarified PPV/NPV calculation methods and added Bayesian projection formulas.
