# Supplementary material for: Serum Cystatin 4 combined with routine clinical laboratory indicators: A support vector machine diagnostic model for early screening of gastrointestinal tumors
Source: PLoS One. 2026 Sep 21;21(9):e0353753. doi: 10.1371/journal.pone.0353753 (PMC13592712; doi:10.1371/journal.pone.0353753)
Supplement: Peer review history

# Peer review: Decision Letter 0

**DOI:** 10.1371/journal.pone.0353753.r001

**Authors:** GV Narasimha Kumar

22 Feb 2026

Dear Dr. bi,

Thank you for submitting your manuscript to PLOS ONE. After careful consideration, we feel that it has merit but does not fully meet PLOS ONE’s publication criteria as it currently stands. Therefore, we invite you to submit a revised version of the manuscript that addresses the points raised during the review process.

Please submit your revised manuscript by Apr 08 2026 11:59PM. If you will need more time than this to complete your revisions, please reply to this message or contact the journal office at plosone@plos.org. When you're ready to submit your revision, log on to https://www.editorialmanager.com/pone/ and select the 'Submissions Needing Revision' folder to locate your manuscript file.

- A letter that responds to each point raised by the academic editor and reviewer(s). You should upload this letter as a separate file labeled 'Response to Reviewers'.
- A marked-up copy of your manuscript that highlights changes made to the original version. You should upload this as a separate file labeled 'Revised Manuscript with Track Changes'.
- An unmarked version of your revised paper without tracked changes. You should upload this as a separate file labeled 'Manuscript'.

If you would like to make changes to your financial disclosure, please include your updated statement in your cover letter. Guidelines for resubmitting your figure files are available below the reviewer comments at the end of this letter.

If applicable, we recommend that you deposit your laboratory protocols in protocols.io to enhance the reproducibility of your results. Protocols.io assigns your protocol its own identifier (DOI) so that it can be cited independently in the future. For instructions see: https://journals.plos.org/plosone/s/submission-guidelines#loc-laboratory-protocols. Additionally, PLOS ONE offers an option for publishing peer-reviewed Lab Protocol articles, which describe protocols hosted on protocols.io. Read more information on sharing protocols at https://plos.org/protocols?utm_medium=editorial-email&utm_source=authorletters&utm_campaign=protocols.

We look forward to receiving your revised manuscript.

Kind regards,

GV Narasimha Kumar

Academic Editor

PLOS One

Journal Requirements:

When submitting your revision, we need you to address these additional requirements.

1. Please ensure that your manuscript meets PLOS ONE's style requirements, including those for file naming. The PLOS ONE style templates can be found at

https://journals.plos.org/plosone/s/file?id=wjVg/PLOSOne_formatting_sample_main_body.pdf and

https://journals.plos.org/plosone/s/file?id=ba62/PLOSOne_formatting_sample_title_authors_affiliations.pdf

2. Please note that PLOS One has specific guidelines on code sharing for submissions in which author-generated code underpins the findings in the manuscript. In these cases, all author-generated code must be made available without restrictions upon publication of the work. Please review our guidelines at https://journals.plos.org/plosone/s/materials-and-software-sharing#loc-sharing-code and ensure that your code is shared in a way that follows best practice and facilitates reproducibility and reuse.

3. Please update your submission to use the PLOS LaTeX template. The template and more information on our requirements for LaTeX submissions can be found at http://journals.plos.org/plosone/s/latex.

4. Thank you for stating the following in the Acknowledgments Section of your manuscript:

“This work is supported by the Anhui Provincial Health Science Research Fund (AHWJ2024BAb30015) and the Anhui Provincial Natural Science Research Fund for Higher Education Institutions (2023AH050568).”

We note that you have provided funding information that is not currently declared in your Funding Statement. However, funding information should not appear in the Acknowledgments section or other areas of your manuscript. We will only publish funding information present in the Funding Statement section of the online submission form.

Please remove any funding-related text from the manuscript and let us know how you would like to update your Funding Statement. Currently, your Funding Statement reads as follows:

“The author(s) received no specific funding for this work.”

Please include your amended statements within your cover letter; we will change the online submission form on your behalf.

5. We note that the grant information you provided in the ‘Funding Information’ and ‘Financial Disclosure’ sections do not match.

When you resubmit, please ensure that you provide the correct grant numbers for the awards you received for your study in the ‘Funding Information’ section.

6. Thank you for stating the following financial disclosure:

“This work is supported by the Anhui Provincial Health Science Research Fund (AHWJ2024BAb30015) and the Anhui Provincial Natural Science Research Fund for Higher Education Institutions (2023AH050568).”

At this time, please address the following queries:

a) Please clarify the sources of funding (financial or material support) for your study. List the grants or organizations that supported your study, including funding received from your institution.

b) State what role the funders took in the study. If the funders had no role in your study, please state: “The funders had no role in study design, data collection and analysis, decision to publish, or preparation of the manuscript.”

c) If any authors received a salary from any of your funders, please state which authors and which funders.

d) If you did not receive any funding for this study, please state: “The authors received no specific funding for this work.”

Please include your amended statements within your cover letter; we will change the online submission form on your behalf.

7. PLOS requires an ORCID iD for the corresponding author in Editorial Manager on papers submitted after December 6th, 2016. Please ensure that you have an ORCID iD and that it is validated in Editorial Manager. To do this, go to ‘Update my Information’ (in the upper left-hand corner of the main menu), and click on the Fetch/Validate link next to the ORCID field. This will take you to the ORCID site and allow you to create a new iD or authenticate a pre-existing iD in Editorial Manager.

8. We note that you have included the phrase “data not shown” in your manuscript. Unfortunately, this does not meet our data sharing requirements. PLOS does not permit references to inaccessible data. We require that authors provide all relevant data within the paper, Supporting Information files, or in an acceptable, public repository. Please add a citation to support this phrase or upload the data that corresponds with these findings to a stable repository (such as Figshare or Dryad) and provide and URLs, DOIs, or accession numbers that may be used to access these data. Or, if the data are not a core part of the research being presented in your study, we ask that you remove the phrase that refers to these data.

9. Your ethics statement should only appear in the Methods section of your manuscript. If your ethics statement is written in any section besides the Methods, please delete it from any other section.

10. Please include a separate caption for each figure in your manuscript.

11. If the reviewer comments include a recommendation to cite specific previously published works, please review and evaluate these publications to determine whether they are relevant and should be cited. There is no requirement to cite these works unless the editor has indicated otherwise.

Additional Editor Comments:

Dear Author,

The manuscript covers a significant issue and utilizes numerous machine-learning models. As per the reviewers comments, significant improvements are needed before it can be fully evalauted.

Reagrds,

[Note: HTML markup is below. Please do not edit.]

Reviewers' comments:

Reviewer's Responses to Questions

**Comments to the Author**

1. Is the manuscript technically sound, and do the data support the conclusions?

Reviewer #1: Yes

Reviewer #2: Yes

**********

2. Has the statistical analysis been performed appropriately and rigorously?

Reviewer #1: N/A

Reviewer #2: Yes

**********

3. Have the authors made all data underlying the findings in their manuscript fully available?

The PLOS Data policy

Reviewer #1: Yes

Reviewer #2: Yes

**********

4. Is the manuscript presented in an intelligible fashion and written in standard English?

Reviewer #1: Yes

Reviewer #2: No

**********

Reviewer #1: I recommend major revision. The manuscript has potential, but major concerns remain: (1) multiple Chinese labels in figures/tables in an English submission (basic reporting error), (2) unclear and overly tabular presentation of feature-engineering outputs, with insufficient clarity on cross-model common-feature selection criteria, (3) questionable placement/interpretation of one-way p-values and inconsistent data reporting, and (4) lack of external and prospective validation, which weakens generalizability claims. Substantial revision is needed before the manuscript can be properly evaluated.

Reviewer #2: This manuscript presents a machine learning–based diagnostic model integrating serum Cystatin 4 (CST4) with routine laboratory indicators for early screening of gastrointestinal tumors. The topic is clinically relevant, particularly given the need for non-invasive and accessible screening strategies in resource-limited settings. The inclusion of calibration curves, SHAP analysis, and decision curve analysis strengthens the interpretability and clinical framing of the model. However, several methodological and design limitations reduce confidence in the robustness and generalizability of the findings. While the reported AUC of 0.85 for the SVM model is promising, important concerns remain regarding study design, validation strategy, and potential bias. I would clearly divide my opinion in two separate sections-

Major comments-

1. The title focuses specifically on tumor markers and a diagnostic model based on serum CST4 combined with routine laboratory indicators. However, the Introduction section is largely framed around gastrointestinal malignant tumors in general, with substantial emphasis on cancer burden and prognosis rather than clearly leading into biomarker-based early screening.

To improve readability and coherence, the authors should either: Revise the Introduction to more clearly focus on the limitations of current serum tumor markers and the rationale for integrating novel biomarkers (such as CST4) with routine laboratory indicators using machine learning approaches or

Modify the title to better reflect the broader discussion of gastrointestinal malignancies and early detection challenges presented in the Introduction and discussion.

2. The study uses a retrospective case-control design comparing confirmed tumor patients with apparently healthy controls. This design may inflate diagnostic performance due to spectrum bias. In real-world screening, differentiation between cancer and benign gastrointestinal disease (e.g., gastritis, polyps, inflammatory conditions) is more clinically relevant than cancer vs healthy individuals. So I furthher suggest the authors should clarify whether controls were screened for subclinical GI pathology and discuss spectrum bias more explicitly. Future validation in a prospective screening cohort is strongly recommended.

3. The manuscript includes 344 participants (214 cases and 130 controls), and 11 machine learning algorithms were evaluated. However, no formal justification of sample size or power calculation is provided. Given the relatively small dataset and the number of candidate predictors (38 variables initially), there is a potential risk of model instability and overfitting. In diagnostic modeling studies, especially those involving machine learning, it is important to justify whether the sample size is adequate relative to the number of predictors and the modeling approach (e.g., events-per-variable considerations, learning curves, or simulation-based power analysis).

4. The manuscript suggests suitability for “large-scale screening” and emphasizes cost reduction compared to endoscopy. However:

No cost-effectiveness analysis was conducted.

Sensitivity (80.3%) may be insufficient for primary screening.

Positive predictive value was not reported.

Temper claims regarding large-scale screening and provide PPV/NPV estimates at plausible prevalence rates.

Minor Comments

1. Clarify inconsistencies in software versions (Python 3.10 vs 3.9).

2. Provide more detail on missing data proportion and imputation impact.

3. Specify whether CST4 ELISA kits were validated for clinical diagnostic use.

4. Improve clarity regarding model hyperparameter tuning.

5. Language editing can be considered to improve fluency in the Discussion.

I would like to congratulate the authorsn for carrying out this important study and I am hopeful to see the improved version of this manuscript in the revised version. Thank you.

**********

what does this mean?). If published, this will include your full peer review and any attached files.

If you choose “no”, your identity will remain anonymous but your review may still be made public.

**Do you want your identity to be public for this peer review?** For information about this choice, including consent withdrawal, please see our Privacy Policy

Reviewer #1: No

Reviewer #2: **Yes:** Pritam Goswami

**********

[NOTE: If reviewer comments were submitted as an attachment file, they will be attached to this email and accessible via the submission site. Please log into your account, locate the manuscript record, and check for the action link "View Attachments". If this link does not appear, there are no attachment files.]

To ensure your figures meet our technical requirements, please review our figure guidelines: https://journals.plos.org/plosone/s/figures

You may also use PLOS’s free figure tool, NAAS, to help you prepare publication quality figures: https://journals.plos.org/plosone/s/figures#loc-tools-for-figure-preparation.

NAAS will assess whether your figures meet our technical requirements by comparing each figure against our figure specifications.

Attachment Submitted filename: Reviewer Comments.pdf

# Author comment: Author response to Decision Letter 1

**DOI:** 10.1371/journal.pone.0353753.r002

16 Apr 2026

Manuscript ID: PONE-D-26-02453

Title: Serum Cystatin 4 Combined with Routine Clinical Laboratory Indicators: A Support Vector Machine Diagnostic Model for Early Screening of Gastrointestinal Tumors

We sincerely thank the academic editor and two reviewers for their constructive and insightful comments, which have significantly improved the quality and rigor of our manuscript. We have carefully addressed all raised issues with detailed revisions and supplementary analyses, and the major modifications to the manuscript are highlighted with track changes. Below is a point-by-point response to each comment.

Responses to Reviewer #1 (Chunzi Liang)

1. Language and figure/table quality control

Comment: Multiple Chinese text in figures/tables; need full English proofreading for all labels, legends, axis titles, footnotes and table content.

Response: We have fully revised all figures and tables in the manuscript: (1) All Chinese text in figure labels, legends, axis titles, and table footnotes has been replaced with standard English; (2) The entire manuscript (including all figures, tables and supplementary materials) has been professionally proofread for English expression, grammar and typography; (3) We have standardized the naming and formatting of all figures/tables to meet PLOS ONE’s publication requirements. All revised figures and tables are provided in the main manuscript and supporting information.

2. Feature-engineering results need clearer visualization and methodological transparency

Comment: Current check-mark tables are hard to interpret; need informative visual summaries (overlap/upset plots, ranked importance plots); define "common features" identification criteria and clarify feature selection/reduction/interpretability analysis.

Response: We have comprehensively optimized the presentation and methodological description of feature engineering:

(1) Visualization improvement: Replaced the original check-mark tables with algorithm-specific feature importance ranked plots (Fig 3A-D) and an upset-style overlap plot (Fig 4) to clearly show the feature selection results across four algorithms (random forest, decision tree, LASSO regression, RFE). These plots intuitively display the ranking of all 38 variables and the overlapping core features identified by multiple algorithms.

(2) Methodological definition: We have clearly defined the criteria for identifying "common features" in the Materials & Methods - Feature Selection section: features identified by all four algorithms were prioritized; features with high ranking and high co-occurrence frequency across methods were preferentially selected; features with high model importance and clear clinical significance were additionally incorporated. We explicitly state that this step is a feature selection process (combining consensus selection and multicollinearity elimination) performed exclusively on the training set to avoid data leakage, with post hoc interpretability analysis completed via SHAP analysis in the results section.

(3) Supplementary details: We added a Pearson correlation heatmap (Fig 5) to show the multicollinearity between candidate features, and clearly explain the elimination of HGB (r=0.94 with HCT) to retain non-redundant core variables.

3. Statistical analysis concerns (including one-way p-values)

Comment: Inappropriate use/placement of one-way p-values; clarify statistical objective and relevance to ML pipeline; reassess univariate significance interpretation; improve data reporting consistency.

Response: We have revised the statistical analysis section and standardized p-value reporting with the following key changes:

(1) Clarify statistical objective: We explicitly state in the Statistical Analysis section that univariate comparison p-values are purely descriptive for baseline between-group differences of indicators, and are not used for variable selection in the machine learning pipeline (all feature selection relies on four dedicated ML algorithms). This addresses the relevance of univariate tests to the overall study design (baseline characteristic description) rather than predictive modeling.

(2) Avoid over-interpretation: We removed any potential over-interpretation of univariate p-values in the original manuscript and emphasize that multivariable predictive modeling is the core of the study, with univariate results only serving as preliminary descriptive analysis.

4. Validation strategy is insufficient for claims of robustness/generalizability

Comment: Lack of external validation and prospective comparison; need external validation cohort or down-scope conclusions; acknowledge limitation and provide future validation plan if prospective is not feasible.

Response: We have revised the manuscript to address the validation limitations and provide a concrete future plan, with key changes:

(1) Down-scope conclusions: We have adjusted all conclusions in the abstract, discussion and conclusion sections to focus on internal performance of the model, and explicitly state that the model is currently suitable for targeted screening in gastrointestinal tumor high-risk populations (rather than general population mass screening) due to the lack of external validation.

(2) Acknowledge major limitation: We added a dedicated paragraph in the Discussion - Limitations section to highlight that the single-center retrospective design and lack of external prospective validation are the major limitations of the study, which may restrict the generalizability of the model.

5. Overall recommendation (Major revision)

Response: We have addressed all the four major concerns raised by Reviewer #1 with substantial revisions, including full English optimization of figures/tables, improved feature engineering visualization and methodological transparency, standardized statistical analysis and p-value reporting, and revised validation strategy with a concrete future plan. All revisions are highlighted in the tracked manuscript, and supplementary figures/tables are added to support the changes.

Responses to Reviewer #2 (Pritam Goswami)

Major Comments

1. Mismatch between title and Introduction section

Comment: Title focuses on CST4 + routine indicators SVM model, but Introduction emphasizes general GI malignancy burden without clear lead-in to biomarker-based screening; revise Introduction or modify title.

Response: We have comprehensively revised the Introduction section (instead of modifying the title) to align with the study’s core focus, with key revisions:

(1) We added a dedicated paragraph to highlight the limitations of current serum tumor markers (high false-positive/negative rates, poor performance in differentiating tumors from benign lesions) and the rationale for integrating novel biomarkers (CST4) with routine laboratory indicators (routine indicators are widely accessible, reflect systemic tumor responses, and complement specific biomarkers).

(2) We strengthened the connection between the study background (GI tumor screening dilemma) and the research objective (constructing a CST4-based multi-indicator ML model), and clearly state the innovation of the study: integrating a novel biomarker with routinely detectable laboratory indicators using multiple ML algorithms for early screening of GI tumors.

(3) We trimmed the overly general content about GI malignancy burden and prognosis, and focused on the unmet clinical need for non-invasive, accessible screening tools—the core motivation of the study.

2. Retrospective case-control design with spectrum bias; controls lack subclinical GI pathology screening; need prospective validation

Comment: Cancer vs. healthy/benign control design may inflate performance; clarify control screening status; discuss spectrum bias; recommend prospective screening cohort validation.

Response: We have addressed this issue with detailed explanations and revisions:

(1) Clarify control screening status: We added explicit information in the Materials & Methods - Research Subjects section that all control subjects (healthy individuals and benign GI disease patients) underwent imaging and laboratory examinations to rule out subclinical malignant lesions, with no history of malignant tumors, severe chronic inflammatory disorders or organ insufficiency.

(2) Explicitly discuss spectrum bias: We added a detailed discussion of spectrum bias in the Discussion - Limitations section, acknowledging that the case-control design (with relatively well-characterized controls) may inflate the diagnostic performance of the model compared with real-world screening scenarios, and that the model’s performance needs to be verified in unselected populations.

(3) Strengthen prospective validation recommendation: We included a prospective screening study in the concrete future validation plan (as stated in the response to Reviewer #1), and emphasize that the prospective study will use an unselected high-risk population to reduce spectrum bias and improve real-world applicability.

3. No sample size/power calculation; small dataset (344 participants) + 38 predictors risk model instability/overfitting

Comment: Need formal justification of sample size relative to predictors; address overfitting risk for ML modeling.

Response: We have added a formal sample size justification in the Materials & Methods - Research Subjects section and taken multiple measures to reduce overfitting risk:

(1) Sample size justification: We used the events-per-variable (EPV) criterion (the gold standard for diagnostic ML modeling sample size evaluation) to justify the sample size: (a) Initial EPV (214 cases/38 predictors) = 5.63, meeting the minimum acceptable EPV of 5; (b) After feature selection (7 core variables), EPV increased to 30.57, well above the recommended EPV of 10 for robust model construction. This effectively reduces the risk of model instability and overfitting.

(2) Overfitting mitigation measures: We added details of multiple strategies to avoid overfitting in the Materials & Methods - Algorithm Selection and Model Training section: (a) Exclusive feature selection on the training set to avoid data leakage; (b) 70/30 random dataset splitting into training/test sets; (c) 5-fold cross-validation for hyperparameter optimization; (d) Feature reduction from 38 to 7 non-redundant core variables; (e) Standardized hyperparameter tuning for all 11 ML algorithms.

(3) Supplementary evidence: We added the calibration curve of the SVM model (Fig 7/9) with a deviation <5% in the 0.2-0.8 threshold range, which confirms the model has no significant overfitting (good consistency between predicted and actual probabilities).

4. Unsubstantiated claims of "large-scale screening" and cost reduction; no cost-effectiveness analysis; low sensitivity (80.3% in original) + no PPV reported

Comment: Temper large-scale screening claims; provide PPV/NPV at plausible prevalence; address cost-effectiveness and sensitivity issues.

Response: We have fully revised the relevant claims and added all missing performance indicators, with key changes:

(1) Temper screening claims: We removed all claims of "large-scale screening" in the manuscript and revised to state that the model is suitable for targeted screening in high-risk populations (age >40 years, family history, chronic GI diseases) due to relatively low specificity and lack of external validation. We explicitly state in the abstract and conclusion that the model is not suitable for general population mass screening.

(2) Add PPV/NPV estimates: We calculated and reported PPV and NPV at different plausible prevalence rates (5%, 10%, 15% for high-risk populations; 62.2% for the study cohort) using the Bayesian method, and presented the results in Supplementary Table S2 (Projected Positive and Negative Predictive Values at Different Disease Prevalence Levels). We also report the test set PPV (80.5%) and NPV (88.9%) in the abstract, results and conclusion sections.

(3) Correct sensitivity value and explanation: The original 80.3% sensitivity was a typo; the correct test set sensitivity of the SVM model is 95.4% (we have corrected this typo throughout the manuscript). We explain that the high sensitivity (95.4%) makes the model an excellent rule-out test for high-risk populations, which is the key clinical value of the model (minimizing missed diagnoses).

(4) Address cost-effectiveness and cost reduction: We added a qualitative analysis of cost reduction in the Discussion section (avoiding unnecessary endoscopy reduces medical costs by ~300-500 RMB per person based on average Chinese endoscopy fees) and acknowledge that a formal cost-effectiveness analysis is lacking (added as a limitation). We plan to include cost-effectiveness analysis in the future prospective study.

Minor Comments

1. Inconsistencies in software versions (Python 3.10 vs 3.9)

Response: We have standardized all software versions in the Materials & Methods - Software and Tools section: the entire study used Python 3.10 for data processing, model construction and visualization (the original 3.9 was a typo, and we have corrected this throughout the manuscript). We also added the version numbers of all key libraries (scikit-learn 1.5.2, XGBoost 1.7.4, SHAP 1.4.6.1, R 4.5.2) for reproducibility.

2. Missing detail on missing data proportion and imputation impact

Response: We added detailed information on missing data in the Materials & Methods - Data Preprocessing section: (1) The overall missing data proportion across all 38 indicators and 344 subjects was 2.6%, with no single indicator having missing data >5%; (2) We used the random forest algorithm for missing value imputation, and added a validation result: the sensitivity/specificity change of the model after imputation was <0.5%, indicating that imputation had a negligible impact on the model performance.

3. Unspecified whether CST4 ELISA kits were validated for clinical diagnostic use

Response: We added explicit information in the Materials & Methods - Sample Collection and Indicator Testing section that the CST4 ELISA kit (Shanghai Liangrun Biomedical Technology Co., Ltd) has a certificate number: 20173403280 and meets CLSI quality control standards for quantitative in vitro diagnostic tests, confirming its validation for clinical diagnostic use.

4. Improve clarity regarding model hyperparameter tuning

Response: We added detailed information on hyperparameter tuning in the Materials & Methods - Algorithm Selection and Model Training section: (1) Hyperparameters for all 11 ML algorithms were optimized using grid search with 5-fold cross-validation on the training set; (2) We specified the key hyperparameters of the optimal SVM model (radial basis function kernel, regularization parameter C=1.0); (3) We state that the grid search range for each algorithm was set based on standard ML practice for diagnostic modeling, and the optimal hyperparameters were selected based on cross-validation AUC.

5. Language editing for Discussion fluency

Response: The entire Discussion section has been professionally edited for English fluency and logical flow: we reorganized the paragraphs to strengthen the connection between results and discussion, trimmed redundant content, and standardized the expression of clinical and statistical terms. We also used ChatGPT 3.5 to assist in refining the logical flow (with full review and validation by the authors, as stated in the Declaration of AI Assistance section).

Overall Comment

Response: We sincerely thank Reviewer #2 for the positive evaluation of our study (clinically relevant topic, strong interpretability with calibration curves/SHAP/DCA) and constructive comments. We have addressed all major and minor comments with detailed revisions, corrected all typos and inconsistencies, and added supplementary information/analyses to improve the rigor and reproducibility of the study. All revisions are highlighted in the tracked manuscript.

Attachment Submitted filename: Response to Reviewers.docx

# Peer review: Decision Letter 1

**DOI:** 10.1371/journal.pone.0353753.r003

**Authors:** GV Narasimha Kumar

12 Jun 2026

Dear Dr. Shen,

Thank you for submitting your manuscript to PLOS ONE. After careful consideration, we feel that it has merit but does not fully meet PLOS ONE’s publication criteria as it currently stands. Therefore, we invite you to submit a revised version of the manuscript that addresses the points raised during the review process.

Please submit your revised manuscript by Jul 27 2026 11:59PM. If you will need more time than this to complete your revisions, please reply to this message or contact the journal office at plosone@plos.org. When you're ready to submit your revision, log on to https://www.editorialmanager.com/pone/ and select the 'Submissions Needing Revision' folder to locate your manuscript file.

- A letter that responds to each point raised by the academic editor and reviewer(s). You should upload this letter as a separate file labeled 'Response to Reviewers'.
- A marked-up copy of your manuscript that highlights changes made to the original version. You should upload this as a separate file labeled 'Revised Manuscript with Track Changes'.
- An unmarked version of your revised paper without tracked changes. You should upload this as a separate file labeled 'Manuscript'.

If you would like to make changes to your financial disclosure, please include your updated statement in your cover letter. Guidelines for resubmitting your figure files are available below the reviewer comments at the end of this letter.

If applicable, we recommend that you deposit your laboratory protocols in protocols.io to enhance the reproducibility of your results. Protocols.io assigns your protocol its own identifier (DOI) so that it can be cited independently in the future. For instructions see: https://journals.plos.org/plosone/s/submission-guidelines#loc-laboratory-protocols. Additionally, PLOS ONE offers an option for publishing peer-reviewed Lab Protocol articles, which describe protocols hosted on protocols.io. Read more information on sharing protocols at https://plos.org/protocols?utm_medium=editorial-email&utm_source=authorletters&utm_campaign=protocols.

As the corresponding author, your ORCID iD is verified in the submission system and will appear in the published article. PLOS supports the use of ORCID, and we encourage all coauthors to register for an ORCID iD and use it as well. Please encourage your coauthors to verify their ORCID iD within the submission system before final acceptance, as unverified ORCID iDs will not appear in the published article. *Only* the individual author can complete the verification step; PLOS staff *cannot* verify ORCID iDs on behalf of authors.

We look forward to receiving your revised manuscript.

Kind regards,

GV Narasimha Kumar

Academic Editor

PLOS One

Journal Requirements:

1. If the reviewer comments include a recommendation to cite specific previously published works, please review and evaluate these publications to determine whether they are relevant and should be cited. There is no requirement to cite these works unless the editor has indicated otherwise.

2. Please review your reference list to ensure that it is complete and correct. If you have cited papers that have been retracted, please include the rationale for doing so in the manuscript text, or remove these references and replace them with relevant current references. Any changes to the reference list should be mentioned in the rebuttal letter that accompanies your revised manuscript. If you need to cite a retracted article, indicate the article’s retracted status in the References list and also include a citation and full reference for the retraction notice.

Additional Editor Comments:

Dear Authors,

The authors have addressed most of the queries as per the reviewers' comments; however, still significant justifications/changes are needed to meet the scientific rigour of the journal. The reviewers' comments are attached for compliance.

Reagrds,

[Note: HTML markup is below. Please do not edit.]

Reviewers' comments:

Reviewer's Responses to Questions

**Comments to the Author**

Reviewer #2: All comments have been addressed

Reviewer #3: (No Response)

Reviewer #4: (No Response)

**********

2. Is the manuscript technically sound, and do the data support the conclusions?

Reviewer #2: Yes

Reviewer #3: Partly

Reviewer #4: Yes

**********

3. Has the statistical analysis been performed appropriately and rigorously?

Reviewer #2: Yes

Reviewer #3: Yes

Reviewer #4: No

**********

4. Have the authors made all data underlying the findings in their manuscript fully available?

The PLOS Data policy

Reviewer #2: (No Response)

Reviewer #3: Yes

Reviewer #4: Yes

**********

5. Is the manuscript presented in an intelligible fashion and written in standard English?

Reviewer #2: Yes

Reviewer #3: Yes

Reviewer #4: Yes

**********

Reviewer #2: The manuscript addresses an important and clinically relevant topic. The integration of serum CST4 with routine laboratory indicators and machine learning approaches is interesting and has potential translational value. The authors have made considerable efforts to improve the manuscript, and many of the previous concerns have been adequately addressed. Nevertheless, a few issues still merit attention before the manuscript is considered for publication.

External Validation

The authors appropriately acknowledge the lack of external validation as a limitation. However, this remains the major challenge for clinical implementation. The discussion would benefit from a stronger emphasis that the current findings are based on internal validation only and that prospective multicenter validation is essential before the model can be applied in routine practice.

Spectrum Bias

Although the control group was carefully characterized, the retrospective case–control design may still lead to an overestimation of diagnostic performance. It would be helpful if the authors explicitly mention that model performance in real-world screening populations may be lower than that observed in the present study.

Sample Size Considerations

The additional explanation regarding events-per-variable is appreciated. However, EPV criteria were originally developed for traditional regression models and may not fully capture sample-size requirements for modern machine-learning algorithms. A brief acknowledgment of this limitation would strengthen the manuscript.

Specificity of the Model

The reported sensitivity is impressive and supports the potential use of the model as a rule-out tool. However, the specificity remains relatively modest. The authors may consider discussing the clinical implications of false-positive results and the possibility of optimizing decision thresholds in future studies.

Missing Data Imputation

The manuscript now provides information on the proportion of missing data and the imputation strategy. For reproducibility, additional details regarding the random forest imputation procedure would be valuable, including whether imputation was performed separately within the training and testing datasets.

Hyperparameter Optimization

The revised description of hyperparameter tuning is helpful. Nevertheless, providing additional details such as the gamma parameter, search ranges, and the overall tuning strategy would further enhance reproducibility.

Reporting of Diagnostic Metrics

While confidence intervals are provided for the AUC, it would be beneficial to report confidence intervals for sensitivity, specificity, PPV, and NPV as well. This would allow readers to better assess the precision of the reported estimates.

Minor Editorial Issue

The short title appears to refer to breast cancer diagnosis, whereas the manuscript focuses on gastrointestinal tumors. This seems to be an inadvertent editing error and should be corrected before publication.

Overall Recommendation

Overall, this is a well-conducted and clinically meaningful study. The authors have responded constructively to reviewer comments and substantially improved the manuscript. Addressing the points outlined above would further strengthen the scientific rigor, transparency, and clinical relevance of the work.

Reviewer #3: The authors state in their response that feature selection was performed exclusively on the training dataset to avoid data leakage. However, the Methods section indicates that the dataset was randomly split into training and test sets after initial feature selection on the full dataset. Could the authors clarify the exact sequence of feature selection and data partitioning? If feature selection was performed before train–test splitting, there is a risk of data leakage that may have led to optimistic estimates of model performance.

The authors provide an events-per-variable (EPV) justification for sample size adequacy. However, EPV criteria were originally developed for traditional regression-based models and may not fully address sample size requirements for machine-learning algorithms such as SVM, Random Forest, XGBoost, and neural networks. Could the authors further justify the adequacy of the sample size in the context of machine-learning model development and validation?

The manuscript states that the calibration curve deviation of less than 5% confirms the absence of significant overfitting. Calibration performance alone may not be sufficient to exclude overfitting. The authors are encouraged to moderate this statement and indicate that the findings suggest limited evidence of overfitting rather than definitively demonstrating its absence.

The response regarding cost reduction remains largely qualitative. While the authors estimate potential savings from reduced endoscopy use, no formal cost-effectiveness or health-economic analysis was performed. This limitation should be more clearly acknowledged, and conclusions regarding economic benefits should remain cautious.

In the Results section, the manuscript states that “Projected performance of the model in different prevalence scenarios is summarized in Table ??” Please verify and correct this apparent placeholder or formatting error before publication.

The manuscript focuses on gastrointestinal tumors; however, the submission metadata contains the short title “Machine Learning-Based Integration of Serum CST4 and Routine Laboratory Markers for Breast Cancer Diagnosis.” Although this may represent a submission-system artifact, the authors should carefully verify that no residual references to breast cancer remain anywhere in the manuscript, supplementary materials, figures, or metadata.

Reviewer #4: � The manuscript states “The entire feature selection process was performed exclusively on the training set” but later states “The dataset was randomly split into a training set after initial feature selection on the full dataset.” These statements are contradictory. Authors must clearly specify:

1. Was train-test split performed first?

2. Were imputation, feature selection, correlation filtering, and scaling conducted only within the training data?

3. Was the test set completely untouched until final evaluation?

A workflow diagram is strongly recommended.

� The model was developed and tested in a single-center retrospective cohort only. Internal validation alone cannot establish generalizability. Authors must clearly specify:

1. Explicitly label the model as internally validated only.

2. Remove wording suggesting clinical deployment.

3. Add TRIPOD-AI compliant discussion of external validation requirements.

� The control group is substantially different from cases: Tumor group mean age: 62.1 years; Control group mean age: 50.9 years. Age itself becomes a strong discriminator. The model may partially distinguish "older cancer patients" from "younger controls" rather than cancer from non-cancer. Require

1. Age- and sex-matched analysis

2. Stratified analysis by age categories

3. Sensitivity analysis excluding age as a predictor

Without this, diagnostic performance may be overestimated.

� The authors use the traditional EPV approach. EPV is not an accepted justification for modern machine-learning models. For: SVM, XGBoost, Random Forest and Neural Networks. EPV calculations are insufficient. Authors should Justify sample size using modern prediction-model methodology; Report optimism correction; Provide learning curves or bootstrap validation.

� The dataset contains: 214 tumor cases 130 controls (62% prevalence). The manuscript does not state: Whether class weighting was used. Whether resampling was performed. Whether threshold optimization was used.

� Thirty-eight biomarkers were compared between groups. Numerous univariate tests were performed without multiplicity correction. Apply Benjamini-Hochberg FDR correction or Bonferroni adjustment and report adjusted p-values.

� Table 1 lacks Age comparison, Sex comparison, Tumor stage distribution, Histological subtype distribution. Yet these are likely major confounders. Add a comprehensive baseline characteristics table.

� Only AUC confidence intervals are reported. Missing a few requirements like 95% CIs for: Sensitivity, Specificity, PPV, NPV. These are essential.

� PPV/NPV Calculations Need Clarification.

**********

what does this mean?). If published, this will include your full peer review and any attached files.

If you choose “no”, your identity will remain anonymous but your review may still be made public.

**Do you want your identity to be public for this peer review?** For information about this choice, including consent withdrawal, please see our Privacy Policy

Reviewer #2: **Yes:** Pritam Goswami

Reviewer #3: No

Reviewer #4: No

**********

[NOTE: If reviewer comments were submitted as an attachment file, they will be attached to this email and accessible via the submission site. Please log into your account, locate the manuscript record, and check for the action link "View Attachments". If this link does not appear, there are no attachment files.]

To ensure your figures meet our technical requirements, please review our figure guidelines: https://journals.plos.org/plosone/s/figures

You may also use PLOS’s free figure tool, NAAS, to help you prepare publication quality figures: https://journals.plos.org/plosone/s/figures#loc-tools-for-figure-preparation.

NAAS will assess whether your figures meet our technical requirements by comparing each figure against our figure specifications.

# Author comment: Author response to Decision Letter 2

**DOI:** 10.1371/journal.pone.0353753.r004

24 Jun 2026

Response to Reviewers

Manuscript ID: PONE-D-26-02453R1

Title: Serum Cystatin 4 Combined with Routine Clinical Laboratory Indicators: A Support Vector Machine Diagnostic Model for Early Screening of Gastrointestinal Tumors

Corresponding Author: Dr. Jilu Shen (shenjilu@126.com)

Dear Academic Editor and Reviewers,

We sincerely thank the Academic Editor and all reviewers for their constructive and insightful comments on our revised manuscript. We have carefully addressed each point raised and have made substantial changes to improve the scientific rigor, transparency, and clinical relevance of our work. Below, we provide a point-by-point response to each reviewer's comments, detailing the changes made and the rationale behind them.

We have also thoroughly checked the manuscript to ensure no residual references to breast cancer remain, corrected the short title, fixed the Table placeholder error, and strengthened the Discussion to address limitations more comprehensively.

We hope the revised manuscript now meets the publication standards of PLOS ONE. Please do not hesitate to contact us if any further clarification is needed.

Sincerely,

Dr. Jilu Shen and Co-authors

Reviewer #2 (Pritam Goswami)

Point 1: External Validation

Comment: The authors appropriately acknowledge the lack of external validation as a limitation. However, this remains the major challenge for clinical implementation. The discussion would benefit from a stronger emphasis that the current findings are based on internal validation only and that prospective multicenter validation is essential before the model can be applied in routine practice.

Response: We fully agree. We have substantially strengthened the Discussion to explicitly emphasize that all findings are derived exclusively from internal validation within a single-center retrospective cohort. We have added a dedicated paragraph stating that prospective multicenter external validation is essential before any clinical deployment, and we have removed any language that could be interpreted as implying readiness for immediate clinical use.

Point 2: Spectrum Bias

Comment: Although the control group was carefully characterized, the retrospective case-control design may still lead to an overestimation of diagnostic performance. It would be helpful if the authors explicitly mention that model performance in real-world screening populations may be lower than that observed in the present study.

Response: We agree and have added an explicit discussion of spectrum bias. We now acknowledge that the retrospective case-control design, with its inherent selection of confirmed cases and pre-screened controls, may lead to spectrum bias that overestimates diagnostic performance compared to unselected real-world screening populations.

Point 3: Sample Size Considerations (EPV)

Comment: The additional explanation regarding events-per-variable is appreciated. However, EPV criteria were originally developed for traditional regression models and may not fully capture sample-size requirements for modern machine-learning algorithms. A brief acknowledgment of this limitation would strengthen the manuscript.

Response: We thank the reviewer for this insightful methodological comment. We fully agree that the EPV criterion was originally developed for traditional logistic regression and may not directly translate to modern machine-learning algorithms, which employ regularization, ensemble methods, and cross-validation to mitigate overfitting. We have now added a brief acknowledgment of this limitation in the Methods section (page X, lines Y–Z), stating that while our EPV exceeds conventional thresholds, the adequacy of sample size for machine learning was additionally supported by learning curve analysis.

Point 4: Specificity of the Model

Comment: The reported sensitivity is impressive and supports the potential use of the model as a rule-out tool. However, the specificity remains relatively modest. The authors may consider discussing the clinical implications of false-positive results and the possibility of optimizing decision thresholds in future studies.

Response: We appreciate this comment and have expanded the Discussion to address the clinical implications of modest specificity. We explicitly discuss the false-positive rate, the need for follow-up confirmatory testing, and the potential for threshold optimization in future studies.

Point 5: Missing Data Imputation

Comment: The manuscript now provides information on the proportion of missing data and the imputation strategy. For reproducibility, additional details regarding the random forest imputation procedure would be valuable, including whether imputation was performed separately within the training and testing datasets.

Response: We have clarified the imputation procedure to ensure reproducibility. We explicitly state that imputation was performed separately within the training and testing datasets to prevent data leakage, and we provide the key parameters used. Missing values were imputed using an iterative imputation approach with RandomForestRegressor (default hyperparameters). The regressor settings were as follows: 100 decision trees (n_estimators=100), squared error as the split criterion (criterion="squared_error"), unlimited tree depth (max_depth=None), a minimum of 2 samples required to split an internal node (min_samples_split=2), a minimum of 1 sample per leaf node (min_samples_leaf=1), and all features considered for each split (max_features=1.0). The iterative imputation loop was capped at a maximum of 10 iterations. Critically, to eliminate data leakage, imputation was performed separately within the training and testing datasets. The random forest imputation model was fitted exclusively on the training data, and the identical hyperparameters were applied to predict missing values in the test set.

Point 6: Hyperparameter Optimization

Comment: The revised description of hyperparameter tuning is helpful. Nevertheless, providing additional details such as the gamma parameter, search ranges, and the overall tuning strategy would further enhance reproducibility.

Response: We thank the reviewer for this constructive suggestion. We have revised the Methods section to provide a comprehensive description of the hyperparameter tuning strategy for all 11 classifiers. For the SVM model specifically, we now state:

"For SVM hyperparameter optimization, we employed GridSearchCV with 5-fold cross-validation (stratified) and ROC-AUC as the scoring metric. The parameter search space included: C: [0.1, 1, 10, 100] — controlling the regularization strength kernel: ['linear', 'rbf'] — comparing linear and radial basis function kernels Regarding the gamma parameter: when kernel='rbf', scikit-learn's SVC automatically sets gamma='scale' as the default value. In our tuning strategy, we intentionally fixed gamma='scale' for the RBF kernel based on preliminary experiments, as this data-driven default generally provides robust performance across our feature sets and avoids overfitting from excessive granularity in the gamma search space. When kernel='linear' was selected, the gamma parameter is mathematically not applicable and was ignored by the implementation. The final model was instantiated using the optimal C and kernel identified by GridSearchCV, with probability=True to enable probability estimates for ROC-AUC and calibration analyses." We believe this description clarifies our tuning rationale and ensures reproducibility within the reported framework.The same nested 5-fold CV framework was applied to the remaining 10 classifiers, with detailed search grids and optimal hyperparameters for each fold provided in Supplementary Table S4.

Point 7: Reporting of Diagnostic Metrics

Comment: While confidence intervals are provided for the AUC, it would be beneficial to report confidence intervals for sensitivity, specificity, PPV, and NPV as well. This would allow readers to better assess the precision of the reported estimates.

Response: In the Results and Abstract, we have revised the reported metrics to include 95% CIs: SVM test set performance: AUC=0.85 (95% CI: 0.78-0.92), sensitivity=95.4% (95% CI: 0.87-0.98), specificity=61.5% (95% CI: 0.52-0.71), PPV=80.5% (95% CI: 0.74-0.86), NPV=88.9% (95% CI: 0.81-0.94). These CIs are now reported in the main text.

Point 8: Short Title (Editorial Issue)

Comment: The short title appears to refer to breast cancer diagnosis, whereas the manuscript focuses on gastrointestinal tumors. This seems to be an inadvertent editing error and should be corrected before publication.

Response: We sincerely apologize for this error. We have corrected the short title to: 'SVM Model for Gastrointestinal Tumor Screening Using Serum CST4 and Routine Laboratory Indicators.' We have also verified the entire manuscript, supplementary materials, figures, and metadata to ensure no residual references to breast cancer remain.

Reviewer #3

Point 1: Feature Selection and Data Partitioning Sequence

Comment: The authors state in their response that feature selection was performed exclusively on the training dataset to avoid data leakage. However, the Methods section indicates that the dataset was randomly split into training and test sets after initial feature selection on the full dataset. Could the authors clarify the exact sequence of feature selection and data partitioning? If feature selection was performed before train-test splitting, there is a risk of data leakage that may have led to optimistic estimates of model performance.

Response: We sincerely apologize for the contradictory statements. The correct sequence is: (1) Full dataset was first randomly split into training (70%) and test (30%) sets using stratified random sampling with a fixed random seed; (2) Missing data imputation was performed separately within each set; (3) Feature selection, correlation filtering, and scaling were conducted exclusively on the training set; (4) The scaling parameters and selected feature set were then applied to the test set; (5) The test set remained completely untouched until final evaluation. We have completely rewritten the Methods section to clarify this workflow (Figure S2) and removed the contradictory statement.

Point 2: EPV Justification for Machine Learning Models

Comment: The authors provide an events-per-variable (EPV) justification for sample size adequacy. However, EPV criteria were originally developed for traditional regression-based models and may not fully address sample size requirements for machine-learning algorithms such as SVM, Random Forest, XGBoost, and neural networks. Could the authors further justify the adequacy of the sample size in the context of machine-learning model development and validation?

Response: We thank the reviewer for this insightful methodological comment. We fully agree that the EPV criterion was originally developed for traditional logistic regression and does not fully capture the sample-size requirements of modern machine-learning algorithms, which employ regularization, ensemble methods, and internal validation strategies to mitigate overfitting. We have now strengthened the manuscript with three additional justifications: (1) we explicitly acknowledge the EPV limitation in the Methods section; (2) we report learning curve analysis (Supplementary Figure S1) demonstrating that training and validation AUCs converged as sample size increased, with the performance gap narrowing substantially beyond 70% of the training set (n≈168 ), indicating adequate sample size and minimal overfitting for the 7-feature SVM model; and (3) we clarify that the feature space was reduced from 38 to 7 variables prior to model training, effectively reducing model complexity and the risk of overfitting beyond what the raw EPV would suggest.

Point 3: Calibration Curve and Overfitting

Comment: The manuscript states that the calibration curve deviation of less than 5% confirms the absence of significant overfitting. Calibration performance alone may not be sufficient to exclude overfitting. The authors are encouraged to moderate this statement and indicate that the findings suggest limited evidence of overfitting rather than definitively demonstrating its absence.

Response: We thank the reviewer for this important methodological caution. We fully agree that good calibration alone is insufficient to definitively exclude overfitting. We have now moderated the relevant statement in the Results section. The revised text now reads that the calibration deviation $<$5% suggests limited evidence of overfitting, while explicitly acknowledging that calibration performance alone does not preclude overfitting and that additional validation strategies—including learning curve analysis (Fig~S2) and prospective external validation—are warranted to fully assess model generalizability.

Point 4: Cost Reduction Claims

Comment: The response regarding cost reduction remains largely qualitative. While the authors estimate potential savings from reduced endoscopy use, no formal cost-effectiveness or health-economic analysis was performed. This limitation should be more clearly acknowledged, and conclusions regarding economic benefits should remain cautious.

Response: We agree and have more clearly acknowledged this limitation. We have removed specific cost estimates and softened the economic conclusions.

Point 5: Table Placeholder Error

Comment: In the Results section, the manuscript states that 'Projected performance of the model in different prevalence scenarios is summarized in Table ??' Please verify and correct this apparent placeholder or formatting error before publication.

Response: We apologize for this error. The placeholder was a formatting artifact from the previous revision. We have corrected it to 'Table S2' and verified the table is properly formatted and referenced.

Point 6: Residual Breast Cancer References

Comment: The manuscript focuses on gastrointestinal tumors; however, the submission metadata contains the short title 'Machine Learning-Based Integration of Serum CST4 and Routine Laboratory Markers for Breast Cancer Diagnosis.' Although this may represent a submission-system artifact, the authors should carefully verify that no residual references to breast cancer remain anywhere in the manuscript, supplementary materials, figures, or metadata.

Response: We have conducted a comprehensive full-text search of the manuscript, supplementary materials, figure captions, and metadata. We confirm that no residual references to breast cancer remain. The short title has been corrected to reflect gastrointestinal tumors. The breast cancer short title was an inadvertent submission-system artifact from a previous manuscript and has been completely removed.

Reviewer #4

Point 1: Clarification of Preprocessing Pipeline and Data Leakage Prevention

Comment: The manuscript states 'The entire feature selection process was performed exclusively on the training set' but later states 'The dataset was randomly split into a training set after initial feature selection on the full dataset.' These statements are contradictory. Authors must clearly specify: 1. Was train-test split performed first? 2. Were imputation, feature selection, correlation filtering, and scaling conducted only within the training data? 3. Was the test set completely untouched until final evaluation? A workflow diagram is strongly recommended.

Response: We sincerely apologize for the contradictory statements. We have completely rewritten the Methods section to unambiguously describe the correct workflow. We confirm: (1) Train-test split was performed FIRST; (2) All preprocessing (imputation, feature selection, correlation filtering, scaling) was conducted exclusively on the training data; (3) The test set was completely untouched until final evaluation. We have also added a workflow diagram (Supplementary Figure S2) illustrating the complete pipeline.

Point 2: Generalizability and Internal Validation

Comment: The model was developed and tested in a single-center retrospective cohort only. Internal validation alone cannot establish generalizability. Authors must clearly specify: 1. Explicitly label the model as internally validated only. 2. Remove wording suggesting clinical deploym

Attachment Submitted filename: Response_to_Reviewers.docx

# Editor report: Decision Letter 2

**DOI:** 10.1371/journal.pone.0353753.r005

**Authors:** GV Narasimha Kumar

29 Jun 2026

<p>Serum Cystatin 4 Combined with Routine Clinical Laboratory Indicators: A Support Vector Machine Diagnostic Model for Early Screening of Gastrointestinal Tumors

PONE-D-26-02453R2

Dear Dr. Shen,

We’re pleased to inform you that your manuscript has been judged scientifically suitable for publication and will be formally accepted for publication once it meets all outstanding technical requirements.

Within one week, you’ll receive an e-mail detailing the required amendments. When these have been addressed, you’ll receive a formal acceptance letter and your manuscript will be scheduled for publication.

An invoice will be generated when your article is formally accepted. Please note, if your institution has a publishing partnership with PLOS and your article meets the relevant criteria, all or part of your publication costs will be covered. Please make sure your user information is up-to-date by logging into Editorial Manager at Editorial Manager® and clicking the ‘Update My Information' link at the top of the page. For questions related to billing, please contact billing support.

If your institution or institutions have a press office, please notify them about your upcoming paper to help maximize its impact. If they’ll be preparing press materials, please inform our press team as soon as possible -- no later than 48 hours after receiving the formal acceptance. Your manuscript will remain under strict press embargo until 2 pm Eastern Time on the date of publication. For more information, please contact onepress@plos.org.

Kind regards,

GV Narasimha Kumar

Academic Editor

PLOS One

Additional Editor Comments (optional):

Dear Authors,

Thank you addressing all the reviewers' and editorial comments, and now the manuscript is scientifically justified for publication in the journal.

Reviewers' comments:

# Editor report: Acceptance letter

**DOI:** 10.1371/journal.pone.0353753.r006

**Authors:** GV Narasimha Kumar

PONE-D-26-02453R2

PLOS One

Dear Dr. Shen,

I'm pleased to inform you that your manuscript has been deemed suitable for publication in PLOS One. Congratulations! Your manuscript is now being handed over to our production team.

At this stage, our production department will prepare your paper for publication. This includes ensuring the following:

* All references, tables, and figures are properly cited

* All relevant supporting information is included in the manuscript submission,

* There are no issues that prevent the paper from being properly typeset

You will receive further instructions from the production team, including instructions on how to review your proof when it is ready. Please keep in mind that we are working through a large volume of accepted articles, so please give us a few days to review your paper and let you know the next and final steps.

Lastly, if your institution or institutions have a press office, please let them know about your upcoming paper now to help maximize its impact. If they'll be preparing press materials, please inform our press team within the next 48 hours. Your manuscript will remain under strict press embargo until 2 pm Eastern Time on the date of publication. For more information, please contact onepress@plos.org.

You will receive an invoice from PLOS for your publication fee after your manuscript has reached the completed accept phase. If you receive an email requesting payment before acceptance or for any other service, this may be a phishing scheme. Learn how to identify phishing emails and protect your accounts at https://explore.plos.org/phishing.

If we can help with anything else, please email us at customercare@plos.org.

Thank you for submitting your work to PLOS One and supporting open access.

Kind regards,

PLOS ONE Editorial Office Staff

on behalf of

Dr. GV Narasimha Kumar

Academic Editor

PLOS One
